# Social tolerance in *Octopus laqueus*—A maximum entropy model

**DOI:** 10.1371/journal.pone.0233834

**Published:** 2020-06-10

**Authors:** Eric Edsinger, Reuven Pnini, Natsumi Ono, Ryoko Yanagisawa, Kathryn Dever, Jonathan Miller

**Affiliations:** 1 Molecular Neurobiology Laboratory, Salk Institute for Biological Studies, La Jolla, CA, United States of America; 2 Josephine Bay Paul Center for Comparative Molecular Biology and Evolution, University of Chicago Marine Biological Laboratory, Woods Hole, MA, United States of America; 3 Okinawa Institute of Science and Technology Graduate University, Onna-son, Okinawa, Japan; 4 Okinawa Enetech, Urasoe City, Okinawa, Japan; 5 Okinawa Fisheries High School, Itoman-shi, Okinawa, Japan; Institut de recherche pour le developpement, FRANCE

## Abstract

*Octopus laqueus* is a small tropical octopus found in Okinawa, Japan and the greater Indo-Pacific. Octopus are often viewed as solitary animals but *O. laqueus* live in close proximity in the wild, and will potentially encounter one another on a regular basis, raising the possibility of social tolerance. Adopting shared den occupancy in aquaria as a potential measure of social tolerance in *O. laqueus*, we studied the animals’ preference for shared dens over solitude. We characterized dependence of sharing preference on sex, den availability and den occupancy density. We designed two simple social tolerance assays in aquaria with a total of 45 daily measurements: (i) Pots Equal, with equal numbers of octopuses and dens and (ii) Pots Limited, with a 3:1 ratio of octopuses to dens. We found that *O. laqueus* will socially tolerate other individuals by sharing tanks and dens and with typically no loss to cannibalism or escape. However, animals also exhibit significant levels of social repulsion, and individuals often chose a solitary den when given the option. The patterns of den occupancy are observed to be consistent with a maximum entropy model that balances seeking shelter against avoiding other animals. The model accurately captures and predicts the data and can be generalized to other organisms and their social interactions. Overall, in *O. laqueus* the preference for a den is stronger than the preference to be solitary. The animals are tolerant of others with a mixture of sizes in the tank and even in a den, a reported first for octopuses outside mating. The relaxed disposition and social tolerance of *O. laqueus* make it a promising species to work with in the lab to explore social and potentially other behaviors in octopuses.

## Introduction

Octopuses are traditionally viewed as solitary animals that do not form social aggregations, have relatively few and simple reciprocal interactions, and rarely make physical contact outside aggression and mating [1–7]. Further, species are known to be cannibalistic in the laboratory and in the field [8–11]. However, recent studies [12–14] suggest that classifying octopus as merely asocial may be simplistic. ‘Asocial’ animals by definition reject or lack the capability for social interaction; they are non-interacting, typically ignoring one another [15]. Some species of octopus exhibit localized aggregated distributions with moderate to high densities depending on factors such as habitat, season, temperature, size, maturity, and prey in the field [1, 14, 16–22], but as Mather observed [1, 23], aggregation alone does not imply sociality.

Octopus aggregations are unlikely to represent gregarious attraction between individuals outside mating [1, 16, 18], but for such aggregations, anti-sociality could yield frequent aggressive or lethal interaction. When individuals encounter one another on a routine basis in a densely localized population, the potential arises for active social interaction, such as touching or visual signaling by body color and patterning.

Social tolerance in dense group cultures in lab has also been reported [24, 25]. It was found, that many animals occupying a single large tank tolerate one another as long as they are well fed and homogeneous in size—a rule of thumb that seems to hold for many octopuses and cephalopods. In addition, octopuses that are largely solitary and asocial in the field can form dominance hierarchies in the lab. In this case, given a set of small dens and a single large den to choose from in group culture, the dominant octopus will take the preferred large den. The shift from solitary to hierarchical social structure in the lab suggests that sociality may be a plastic trait in octopuses, one that is flexible or dependent on the conditions at hand, including population density [1, 7, 26–28].

**Fig 1:**
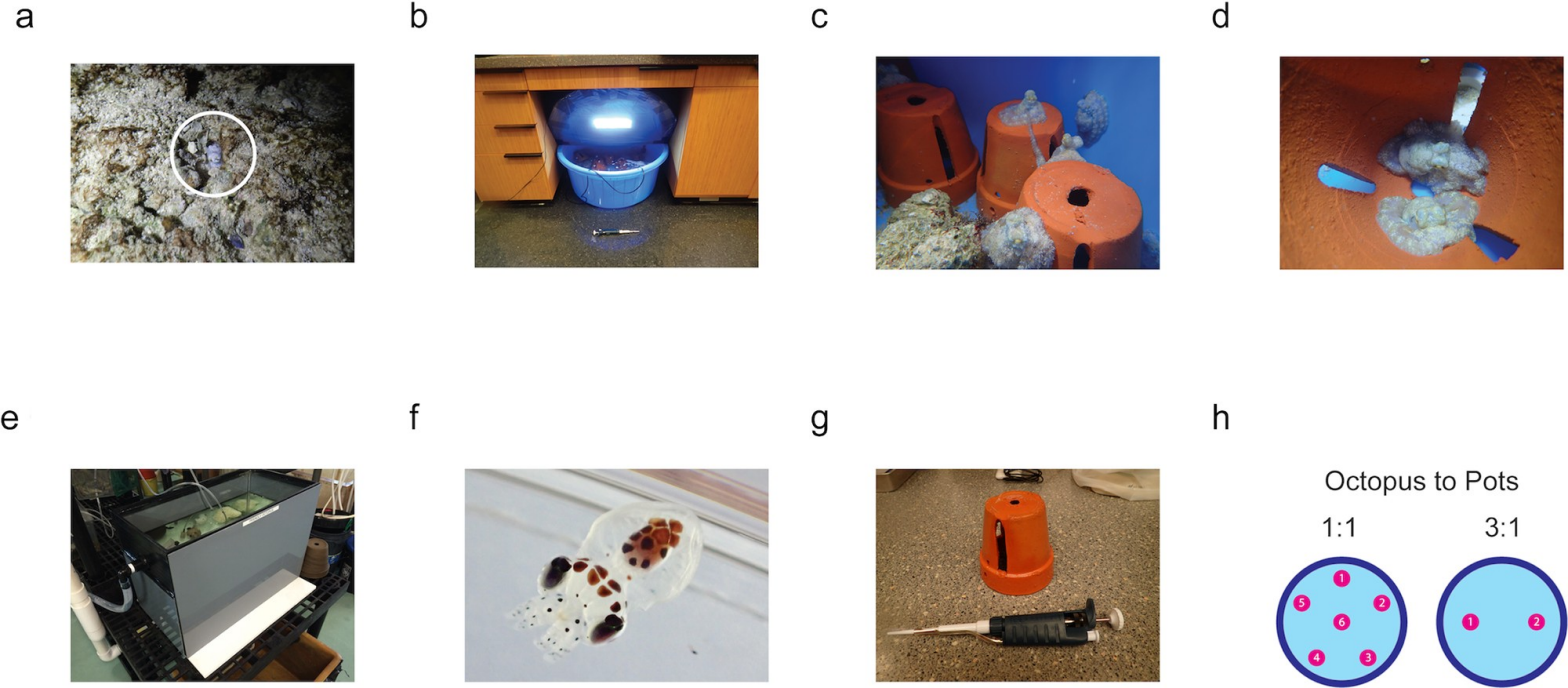
Culturing and tank design of *O. laqueus* in the lab. (a) An adult *O. laqueus* peeking out of its den at Maeda Flats, Okinawa, Japan, where animals were collected for lab culture. (b) An experimental culture tank at OIST with air line, tank cover, LED lights, and clay pots visible. (c) Several *O. laqueus* in a tank in the lab. (d) Two *O. laqueus* sharing a single pot as a den during the day. (e) The long-term culture tank at the MBL. (f) An *O. laqueus* hatchling of a female that was long-term cultured at the MBL, from juvenile through sexual maturity and ending with a natural death by senescence after hatching of her embryos. (g) A single clay pot with a pipettor for scale. (h) Layout of clay pots in the experimental tanks for the social experiments.

A small, shallow-water tropical species, *Octopus laqueus* is common in sand, reef rubble, and reef habitats in Okinawa, Japan, and may be distributed more generally in the tropical Indo-Pacific [2, 29]. We often observed animals within a few meters or less of one another in holes or dens in the sand and reef rubble (Fig 1a) (S1 Video), suggesting that a given individual is likely to encounter multiple conspecifics on a given night of foraging and hunting. The abundance and proximity of *O. laqueus* in the field raised the possibility that it is a social octopus, leading us to wonder whether *O. laqueus* would tolerate conspecifics in a den or tank under dense conditions, and to design a series of experiments to investigate this question.

Our aim is to investigate functional and *predictive*—as opposed to descriptive—characterisations of sociality. Following Mather [15, 30] we define as ‘asocial’ non-interacting animals, e.g. animals that ignore each other in a specified context. The typical number of such animals that share common dens sets a reference level for neutrality. Animals showing levels of sharing larger than neutral are then considered as ‘social’ whereas animals showing smaller levels of sharing are ‘anti-social’(note, that by taking neutral animals as a reference, animals that actively avoid each other would be labeled as anti-social, rather than just being asocial; on the other hand, animals that tolerate each other, yet at levels comparable with the neutral ones, would still be considered as asocial). This operational definition of sociality may not necessarily correspond directly to customary notions of sociality.

The context for our experiments is den occupation patterns. The statistical significance of pot occupancy patterns is established hypothesis-testing methods, with the neutral model serving as a null hypothesis, and by testing statistical contrasts under different experimental treatments. We also develop a statistical model of den occupation based on the maximum entropy principle. The proposed max-ent model transcends hypothesis testing: (i) It treats different experimental setups on the same footing, incorporating together all the measurements and hence increase their statistical power, irrespective of whether these setups are ‘un-balanced’ or ‘un-factorized.’ (ii) It in principle enables compilation of meta-data from diverse labs, future measurements, even from different species or eco-systems into a common framework, in which the statistical uncertainties are explicitly stated. (iii) It offers a natural phenomenological explanation for the social behavior in terms of animal (pairwise) interactions. ‘Natural’ here means a straightforward and simple explanation—using the least structured model, i.e., the one with a minimal set of assumptions which is still consistent with the measurements. (iv) It facilitates verifiable quantitative predictions that *may* apply outside the measured regime. It is therefore useful for designing new experiments, identifying outliers, and exploring potentially interesting experimental regimes. We can project the trade-off between denser populations and mixed sexes for arbitrary numbers of animals and dens.

## Methods

### Ethical considerations

The research adhered to ASAB/ABS Guidelines for the Use of Animals in Research, in addition to legal and institutional requirements in Japan and the United States. Collection, care, and export of many small non-commercial octopus species, including *O. laqueus*, are not regulated in Japan, and permits or licenses from a granting authority were not required. Import of *O. laqueus* from Okinawa to the Marine Biological Laboratory (MBL) in the United States was done in accordance with all applicable US Customs and US Fish and Wildlife regulations. Care of invertebrates like *O. laqueus* does not fall under United States Animal Welfare Act regulation, and is omitted from the PHS-NIH *Guide for the Care and Use of Laboratory Animals*. Thus, an Institutional Animal Care and Use Committee, a Committee on Ethics for Animal Experiments, or other granting authority does not formally review and approve experimental procedures on and care of invertebrate species *O. laqueus* at the MBL. However, in accordance with MBL Institutional Animal Care and Use Committee guidelines for invertebrates, our care and use of *O. laqueus* in Japan and in the United States generally followed tenets prescribed by the Animal Welfare Act, including the three “R’s” (refining, replacing, and reducing unnecessary animal research), and also generally adhered to recent EU regulations and guidelines on the care and use of cephalopods in research [31].

### Collection

*O. laqueus* were collected at night on low tides close to shore in water five to fifty centimeters deep in Okinawa, Japan from Octobrer 2014 to February 2015 and in November 2015. The animals were commonly seen in holes or dens in sand and reef rubble (Fig 1a) and were often observed within a few meters or less of one another, suggesting that each individual is likely to encounter other individuals on a night of foraging and hunting. On three occasions while diving or intertidal walking, sets of two octopus were observed in dens or holes sufficiently nearby for the animals to touch one another, and it was possible that they were sharing a single den with multiple entrances (S1 Video). As a practical matter, animals can be collected only in the winter, by reef-walking at lowest tides that occur at most once or twice a month, over short intervals of time wherein many participating collectors must act simultaneously without the chance to coordinate their efforts until they return to shore when the tide begins to rise. Subject to these conditions, *O. laqueus* were easily caught when found outside the den. Typically 5-10 animals were placed in a single bucket with seawater during collection (Fig 1a). To allow acclimation to the laboratory environment, behavioral experiments in tanks did not begin until several days after collection and the onset of feeding.

### Tolerance in buckets

To obtain a rough indication of whether *O. laqueus* might be socially tolerant, five replicates of around ten octopuses were placed in ten-liter buckets with several liters of seawater and without lids over the course of collection in the field. Octopuses were left in buckets for one to four hours and observed periodically. In our experience, many species of octopus immediately start trying—often successfully—to climb out of the buckets.

### General culturing

*O. laqueus* was cultured at the Okinawa Institute of Science and Technology (OIST), where clay pot experiments described below were performed, and at the MBL, where a long-term culturing experiment described below was performed. In contrast to most octopus species that in our experience must be singly cultured to prevent fighting or cannibalism, *O. laqueus* were easy to care for in *group* cultures in the lab (Fig 1). Animals in OIST were kept at densities up to one animal per 15 liters with four to fifteen animals in 250 liter tanks with filtration, air, and closed circulation. 10-100% of seawater was refreshed every 1-3 days and water quality was checked periodically (pH, nitrates, nitrites, ammonia). Prime (*Seachem*) was periodically used to help stabilize conditions for short-term cultures (days to weeks). For a longer-term group culture of several months, three young juveniles (one male and two females, each around ten grams) and freshly collected were shipped from OIST in Okinawa, Japan to the MBL in Woods Hole, MA, United States. At the MBL, animals were maintained together in a 75-liter aquarium and a sand-filtered flow-thru seawater system. Animals appeared surprisingly relaxed, and were kept in open tanks without lids or deterrents at both OIST and the MBL (Fig 1b and 1e). *O. laqueus* brought into the lab typically began eating within one to two days after collection and accepted freshly killed or store-bought frozen shrimp and crabs without training, in addition to live prey. Seawater at OIST was at room temperature, 21°C; at the MBL temperature was maintained at 23°C, as room temperature was much lower.

### Identification

Animals were visually identified to species [29] and weighed. Sex identifications were also made based on male curling of the right third arm while moving, and on the presence of two large suckers at the proximal end of the arms in males but not females. For identification, octopuses were tagged with silicone-based fluorescent elastomer (*Northwest Marine Technology*) that was injected into a small area in the dorsal mantle [2, 32] (S2 Video). Because of its potential adverse effect on behavior in days after treatment and due to the risk of mortality, anesthesia was not used. Injected octopuses seemed lethargic immediately after injection but recovered within a few hours or by the next morning. Experiments were not begun until several days after injection to ensure all animals had recovered and were behaving normally.

### Social behavior experiments

For social behavior experiments, tagged animals were sorted into four groups of five or six, and each group was cultured in one of four identical circular tanks, with a balance of mixed sizes and sex across tanks of the same treatment when possible (S1–S4 Tables). Animals were maintained on an 11:13 hour light-dark cycle that roughly matched the local light cycle in Okinawa in late November and early December. The tanks were loosely covered with light-proof lids at the onset of the dark cycle to keep out most indoor light, but very dim light was admitted by the mostly but not fully opaque plastic sides of the tank, roughly approximating nocturnal natural light (Fig 1b). Small clay pots (15 cm tall) were used as dens in the tanks. Each pot had 4 large slits along the sides and a hole on top, allowing animals to readily enter and leave a pot and monitor activity outside it (Fig 1c). Clay pots and tanks were scored for animals three hours after the start of the light cycle (Fig 1d). Individuals were identified based on their elastomer tags and their health was generally assessed at this time. To minimize stress from repeated handling, animals were transferred to small individual feeding containers immediately after assessment but prior to feeding. The containers (Critter Keepers) included *very* small clay pots (5 cm tall) as dens. The containers were returned to the larger tanks after the animals were added. One hour prior to the dark cycle, the feeding containers with animals were moved to the bench top and two live or frozen shrimp or crab were added to each container. At the start of the dark cycle, the small containers were covered to block room lighting. A few hours after adding food to the containers, animals were returned to their main tanks to roam freely. This procedure ensured that each animal was equally and adequately fed and prevented fouling of the main tanks from left-over food, which rotted quickly in the warm conditions.

To quantify social tolerance versus social repulsion through pot occupancy in communal tanks, two social behavior treatments were performed with the experimental setup described above: “Pots Equal” (PE) and “Pots Limited” (PL). To balance sizes, each tank included one to two large, three medium, and one small animal. Ranges for the three sizes classes were determined based on the distribution of animal weights across sexes. The PE treatment was preformed in three configurations: mixed sexes (FM), all-female (FF) and all-male (MM). (i) In the FM case, an equal number of octopuses and pots were placed in a tank, and pot and tank occupancy was scored daily for five or six days, with two replicates, twelve octopus, and eleven tank assessments in total. The male:female ratio was 1:1 in all FM replicates (Table 1). (ii) In the PL treatment, two or three octopus per pot were placed in a tank, and pot and tank occupancy was scored daily for seven days, with two replicates, twelve octopus (one octopus was replaced after Day 1 of the second replicate), and fourteen tank assessments in total (Table 2). The male:female ratio was 1:1 in the first replicate, but 1:2 in the second replicate because only a limited number of animals was available. (iii) Using the same animals, potential sex-based differences in social tolerance of shared den occupancy were subsequently tested in a second round of PE treatment, with two all-female (FF) replicates scored daily for five days (twelve octopus and ten tank assessments in total (Table 3) and two all-male (MM) replicates scored daily for five days (eleven octopus and ten tank assessments in total (Table 4). Re-sampling of individuals for testing sex-based differences brought some novel animals together within a single tank for the first time, and also meant that not all octopus could be tracked individually, because there were not enough elastomer colors to label each octopus uniquely.

**Table 1:** Occupation numbers for mixed sexes, *K* = 6 animals in *N* = 6 pots (*K*_*f*_ = *K*_*m*_ = 3). (a) and (b) are two replicas with equal numbers of females and males. *S*_*d*_ = ∑_*i*_ *n*_*i*_ *I*[*n*_*i*_ − 2] is the daily sharing level and *σ*_*ff*_, *σ*_*mm*_, *σ*_*fm*_ are, respectively, the number of female-female, male-male and female-male pairs [Eq (14)]. Bottom lines—the mean values.

		Female: Male occupation numbers							Sharing	#links		
Tank	Day	*n*_0_	*n*_1_	*n*_2_	*n*_3_	*n*_4_	*n*_5_	*n*_6_	*S*_*d*_	*σ*_*ff*_	*σ*_*mm*_	*σ*_*fm*_
**(a)**												
T1	1	0:0	0:1	0:1	1:0	0:1	1:0	1:0	0	0	0	0
T1	2	0:0	1:1	1:0	0:1	1:0	0:1	0:0	2	0	0	1
T1	3	0:0	1:0	1:0	0:1	0:1	0:1	1:0	0	0	0	0
T1	4	0:0	1:0	1:1	0:1	0:0	0:1	1:0	2	0	0	1
T1	5	0:0	0:1	0:1	1:0	1:0	0:1	1:0	0	0	0	0
T1	6	0:0	1:0	0:1	0:1	0:1	0:0	2:0	2	1	0	0
		0:0							6/6	1/6	0	2/6
**(b)**												
T3	1	1:0	1:1	0:0	1:0	0:0	0:1	0:1	2	0	0	1
T3	2	0:0	0:0	1:0	0:1	1:0	1:1	0:1	2	0	0	1
T3	3	0:0	0:0	0:1	0:1	1:0	1:1	1:0	2	0	0	1
T3	4	0:0	0:1	1:1	0:0	0:0	0:1	2:0	4	1	0	1
T3	5	0:0	0:1	1:0	1:1	0:0	0:0	1:1	4	0	0	2
		$\frac{1}{5}$:0							14/5	1/5	0	6/5

**Table 2:** Occupation numbers for mixed sexes, *K* = 6 animals in *N* = 2 pots. (**a**) *K*_*f*_ = *K*_*m*_ = 3 (**b**) *K*_*f*_ = 4, *K*_*m*_ = 2. *S*_*d*_ = ∑_*i*_ *n*_*i*_ *I*[*n*_*i*_ − 2] is the daily sharing level and *σ*_*ff*_, *σ*_*mm*_, *σ*_*fm*_ are, respectively, the number of female-female male-male and female-male pairs [Eq (14)]. Bottom lines—mean values.

		Female: Male			Sharing	#links		
Tank	Day	*n*_0_	*n*_1_	*n*_2_	*S*_*d*_	*σ*_*ff*_	*σ*_*mm*_	*σ*_*fm*_
**(a)**								
T2	1	1:0	1:1	1:2	5	0	1	3
T2	2	0:0	1:1	2:2	6	1	1	5
T2	3	1:0	1:1	1:2	5	0	1	3
T2	4	1:0	1:2	1:1	5	0	1	3
T2	5	1:0	0:2	2:1	5	1	1	2
T2	6	1:1	1:1	1:1	4	0	0	2
T2	7	0:0	1:1	2:2	6	1	1	5
		$\frac{5}{7}$:$\frac{1}{7}$			36/7	3/7	6/7	23/7
**(b)**								
T4	1	1:0	1:0	2:2	4	1	1	4
T4	2	1:0	1:1	2:1	5	1	0	3
T4	3	1:0	2:1	1:1	5	1	0	3
T4	4	1:0	1:1	2:1	5	1	0	3
T4	5	1:0	1:1	2:1	5	1	0	3
T4	6	0:1	2:1	2:0	5	2	0	2
T4	7	0:1	2:0	2:1	5	2	0	2
		$\frac{5}{7}$:$\frac{2}{7}$			34/7	9/7	1/7	20/7

**Table 3:** Occupation numbers for equal number of pots and animals—all females in two replicas (a) and (b). *n*_0_ indicates the number of outsiders that stay out of the pots. *S*_*d*_ = ∑_*i*_ *n*_*i*_ *I*[*n*_*i*_ − 2] is the daily sharing level and *σ*_*ff*_ = ∑_*i*_ *n*_*i*_(*n*_*i*_ − 1)/2 is the number of female-female pairs. bottom lines—the mean values.

Females		occupation numbers							Sharing	#links
Tank	Day	*n*_0_	*n*_1_	*n*_2_	*n*_3_	*n*_4_	*n*_5_	*n*_6_	*S*_*d*_	*σ*_*ff*_
**(a)**										
T3	1	0	1	0	2	1	2	0	4	2
T3	2	0	2	2	1	1	0	0	4	2
T3	3	0	2	1	1	0	1	1	2	1
T3	4	0	2	1	0	0	2	1	4	2
T3	5	0	1	2	0	1	1	1	2	1
		0							16/5	8/5
**(b)**										
T4	6	0	0	0	2	1	1	2	4	2
T4	7	0	0	2	1	1	1	1	2	1
T4	8	0	1	1	1	1	1	1	0	0
T4	9	0	1	1	1	1	1	1	0	0
T4	10	0	1	3	0	0	2	0	5	4
		0							11/5	7/5

**Table 4:** Occupation numbers for equal number of pots and animals—all males in two replicas (a) and (b). *n*_0_ indicates the number of outsiders, *S*_*d*_ = ∑_*i*_ *n*_*i*_ *I*[*n*_*i*_ − 2] is the daily sharing level and *σ*_*mm*_ = ∑_*i*_ *n*_*i*_(*n*_*i*_ − 1)/2 is the number of male-male pairs. Bottom lines—the mean values.

Males		occupation numbers							Sharing	#links
Tank	Day	*n*_0_	*n*_1_	*n*_2_	*n*_3_	*n*_4_	*n*_5_	*n*_6_	*S*_*d*_	*σ*_*mm*_
**(a)**										
T1	1	0	1	1	1	1	1	-	0	0
T1	2	0	1	1	2	0	1	-	2	1
T1	3	0	1	1	0	2	1	-	2	1
T1	4	0	1	1	1	1	1	-	0	0
T1	5	0	1	1	1	1	1	-	0	0
		0							4/5	2/5
**(b)**										
T2	6	0	1	1	1	1	1	1	0	0
T2	7	0	2	0	1	1	1	1	2	1
T2	8	0	1	1	1	1	1	1	0	0
T2	9	0	1	1	1	1	1	1	0	0
T2	10	0	1	1	1	1	1	1	0	0
		0							2/5	1/5

Specific details and data per treatment, experiment, tank, pot, and octopus are available in the Supplemental Materials (S1–S4 Tables). Additional details regarding methods used to perform the social behavior experiments are available here: http://dx.doi.org/10.17504/protocols.io.w9nfh5e.

### Experimental design and statistical power

To estimate statistical power, we defined a daily sharing-level *S*_*d*_(*N*, *K*) for *K* identical animals distributed in *N* pots. Thus, denoting the number of animals in pot *i* by *n*_*i*_ (*i* = 1, 2, …, *N*), the number of animals is $K=\sum_{i=0}^{N}n_{i}$ where *n*_0_ is the number of ‘outsiders’, i.e., animals that remain inside the tank but outside all pots. The sharing-level is defined as

$$\begin{array}{c} S_{d}\left(N,K\right)\equiv \sum_{i=1}^{N}n_{i}I\left[n_{i}-2\right] \end{array}$$ (1)

where the indicator *I* vanishes for *n*_*i*_ = 0, 1 (for a list of symbols cf. Table 5). We estimate the average sharing-level ${\bar{S}}_{d}\left(N,K\right)$ and compare this quantity to a model of neutral animals (the null hypothesis) as a function of the number of independent samples *M* [33].

**Table 5:** List of symbols.

Symbol	Description
*N*	# pots.
*K*	# animals: [*K*_*f*_—females, *K*_*m*_—males].
*M*	# independent observations.
*n*_0_	# ‘outsiders’ that stay in the tank, outside of all pots.
*n*_*i*_	#animals found in pot number *i*(*i* = 1, 2, …, *N*).
$\vec{n}$	configuration, occupation numbers $\vec{n}=\left\{n_{0},n_{1},\ldots ,n_{N}\right\}$.
Ω	total # of configurations.
*g*	multinomial coefficient, $g\left(\vec{n}\right)\equiv \left(n_{0}+n_{1}+\cdots +n_{N}\right)!/\left(n_{1}!n_{1}!\cdots n_{N}!\right)$.
*S*_*d*_	daily sharing level, defined in Eq (1).
*S*_0_	mean sharing level of identical neutral animals, Eq (2).
*σ*	linkage = total # of pairs as defined in Eq (14): [*σ*_*ff*_ female:female, *σ*_*mm*_ male:male, *σ*_*fm*_ female:male]
*μ*	chemical potential.
*U*	on-site interaction: [*U*_*ff*_ female:female, *U*_*mm*_ male:male, *U*_*fm*_ female:male]
*H*	Hamiltonian, energy functional, Eqs (4) and (10).
*Z*	partition function. *F* ≡ − log *Z* is the free energy.
*P*	probability distribution. $P\left(\vec{n}\right)=g\left(\vec{n}\right)e^{-H}/Z$ is the canonical distribution.
〈*x*〉	ensemble averaging of a quantity *x* with respect to the distribution *P*.
〈*δxδy*〉	correlation function of *x* and *y* (connected), *δx* ≡ *x* − 〈*x*〉.
$\bar{x}$	empirical averaging of *x*.
$\hat{x}$	an estimator of a random variable *x*.
*S*[*P*]	entropy, *S*[*P*] = −〈log *P*〉.

The *neutral model* assumes that (i) animals completely ignore each other and therefore can be treated as independent non-interacting particles—volume-fraction of octopus is neglected; and (ii) no animal remains outside a pot, *n*_0_ ≡ 0. One can then think of distributing the animals into pots as rolling an *N*-sided dice *K* times. For identical but ‘distinguishable’(i.e., labeled) animals, the probability of obtaining a specified configuration {*n*_1_, *n*_2_, …, *n*_*N*_} of such neutral animals is given by the multinomial distribution

$$\begin{array}{c} P\left(n_{1},n_{2},\ldots ,n_{N}\right)=K!/\left(n_{1}!n_{2}!\cdots n_{N}!\right)N^{-K} \end{array}$$ (2)

**Fig 2:**
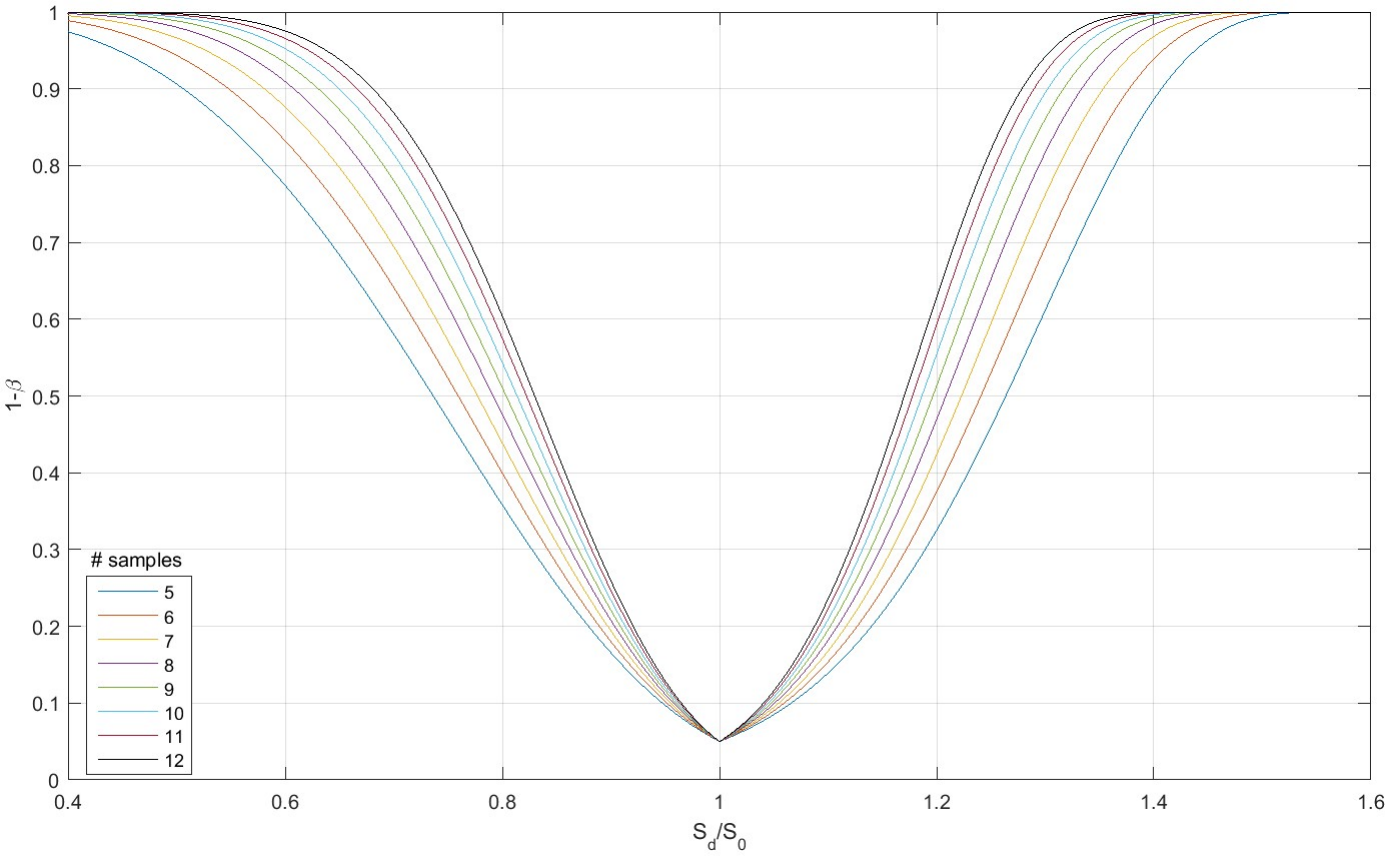
Statistical power vs. relative sharing level for K = 6 animals in N = 6 dens (*S*_0_ = 3.59, *α* = 0.05). *S*_0_ is the average sharing level of the neutral model. *α* and *β* are the probabilities of the type-I and type-II errors, respectively.

Denoting the type-II error by *β*, we set the probability of type-I error *α* to 0.05 and calculate the expected power (1 − *β*) as a function of the ratio ${\bar{S}}_{d}/S_{0}$, where *S*_0_ is the average sharing-level of the neutral model. The distribution of the sample-mean of *M* observations, ${\hat{S}}_{d}=M^{-1}\sum_{m=1}^{M}S_{d}\left(m\right)$, is assumed to be Gaussian with moments *m*_1_ = *Nθ*[1 − (1 − *θ*)^*N*^] and *m*_2_ = [*N*(*N* − 1)*θ*^2^ + *m*_1_]/*M*, where the parameter 0 ≤ *θ* ≤ 1 is determined by setting $m_{1}={\bar{S}}_{d}$. The results for *N* = *K* = 6 (*S*_0_ = 3.59), are shown in Fig 2. It then follows, that *M* = 5 samples are sufficient to detect a deviation from neutrality in the range ${\bar{S}}_{d}\leq 0.55S_{0}$ (anti-social) and ${\bar{S}}_{d}>1.35S_{0}$ (social) at a power of more than 80%.

### Modeling pot occupancies

In the following we obtain a statistical description of pot occupancies that extends beyond neutrality. We employ a maximum entropy principle [34] wherein which distributions and correlations, such as the probability distribution of sharing, are derived from a Hamiltonian. Maximum entropy yields the least structured model consistent with the empirical observations, while quantitatively recapitulating the hypothesis-testing values. Our model might be called the “housemate model” you will let me share your home only if I get along with everyone else in the house; similarly, everyone already living there must get along with one another. Such pairwise propensities, affinities, or proclivities are taken to be independent of and uncorrelated with one another. The probability that *n*_*i*_ agents get along with each other decays exponentially with the total number of pairwise interactions between, or distinct pairs of, agents within pot *i*, *n*_*i*_(*n*_*i*_ − 1)/2. Of course the model is simplistic, but with readily achievable values of *n*_*i*_, it could be misleading to try to fit a model with more parameters or degrees of freedom.

We first study the max-ent model with single-sex distribution. Assuming *K* identical animals distributed among *N* pots, a configuration of animals up to labeling is completely determined by specifying a set of occupation numbers: $\vec{n}=\{n_{0},n_{1},\cdots ,n_{N}\}$. The mean number of outsiders averaged over *M* days,

$$\begin{array}{c} {\bar{n}}_{0}=\frac{1}{M}\sum_{m=1}^{M}n_{0}\left(m\right) \end{array}$$ (3a)

and the mean number of pair-interactions,

$$\begin{array}{c} \bar{\sigma }=\frac{1}{2M}\sum_{m=1}^{M}\sum_{i=1}^{N}n_{i}\left(m\right)\left[n_{i}\left(m\right)-1\right] \end{array}$$ (3b)

are measured experimentally. The probability distribution, $P(\vec{n})$, that maximises the entropy $S\left[P\right]\equiv -\sum_{\vec{n}}P\left(\vec{n}\right)\;\text{log}\;P\left(\vec{n}\right)$ under the empirical constraints $\left({\bar{n}}_{0},\bar{\sigma }\right)$ is the canonical distribution $P\left(\vec{n}\right)=g(\vec{n})\;\text{exp}\;\left[-H\left(\vec{n}\right)\right]/Z$, where

$$\begin{array}{c} H\left(\vec{n}\right)=\mu n_{0}+\frac{U}{2}\sum_{i=1}^{N}n_{i}\left(n_{i}-1\right) \end{array}$$ (4)

is the Hamiltonian, *Z* is the partition function, obtained by summing over all configurations, $Z\left(\mu ,U\right)=\sum_{\vec{n}}g\left(\vec{n}\right)\;\text{exp}\;\left[-H\left(\vec{n}\right)\right]$ and $g\left(\vec{n}\right)\equiv \left(n_{0}+n_{1}+\cdots +n_{N}\right)!$/(*n*_0_!*n*_1_!⋯*n*_*N*_!) is the multinomial coefficient [Eq (2)]. Eq (4) is similar to the bosonic Hubbard model which is well known in condensed matter physics [35]. The parameters introduced in (4) are the on-site (or “contact”) interaction *U*, which can be attractive (*U* < 0) or repulsive (*U* > 0), and the chemical potential *μ* which penalizes the outsiders (*μ* > 0). The chemical potential is the simplest one-body (linear) contribution to a model and the interaction term is quadratic. Positive values of *μ* and *U* describe together a balance between staying inside a den because the open environment is unfavorable, and staying out of a den in order to avoid repulsive den-mates. When *U* = 0 and *μ* → ∞, one recovers the neutral model as a special case of Eq (4).

The parameters in Eq (4) are found by imposing the conditions

$$\begin{array}{c} {\bar{n}}_{0}=\sum_{\vec{n}}P\left(\vec{n}\right)n_{0}\;,\;\bar{\sigma }=\sum_{\vec{n}}P\left(\vec{n}\right)\frac{1}{2}\sum_{i=1}^{N}n_{i}\left(n_{i}-1\right) \end{array}$$ (5)

so that empirical-averaging coincides with ensemble-averaging with respect to $P(\vec{n})$. Namely [here and in the sequel $\bar{x}$ stands for the sample-averaging over a quantity *x* whereas 〈*x*〉 is the ensemble-averaging],

$${\bar{n}}_{0}=-\partial \;\text{log}\;Z/\partial \mu \;,\;\bar{\sigma }=-\partial \;\text{log}\;Z/\partial U$$ (6)

Alternatively, the parameters can be obtained by the maximum likelihood condition,

$$\begin{array}{c} \underset{\left(\mu ,U\right)}{\text{max}}\;W\left(\mu ,U\right)=\underset{\left(\mu ,U\right)}{\text{max}}\sum_{m=1}^{M}\;\text{log}\;P\left[\vec{n}\left(m\right);\mu ,U\right] \end{array}$$ (7)

where $\vec{n}\left(m\right)$ {*m* = 1, 2, …, *M*} are the observed configurations, and log *P* = −(*H* + log *Z*). The error in estimating the parameters is then given by the Gaussian fluctuation [36] (a.k.a. Fisher information matrix), evaluated at the maximum-likelihood solution (*μ*_0_, *U*_0_):

$$\begin{array}{c} {}[\begin{array}{cc} \left\langle \delta {\hat{\mu }}^{2}\right\rangle & \left\langle \delta \hat{\mu }\delta \hat{U}\right\rangle \\ \left\langle \delta \hat{U}\delta \hat{\mu }\right\rangle & \left\langle \delta {\hat{U}}^{2}\right\rangle \end{array}{]}^{-1}=M(\begin{array}{cc} \partial^{2}\;\text{log}\;Z/\partial \mu^{2} & \partial^{2}\;\text{log}\;Z/\partial \mu \partial U\\ \partial^{2}\;\text{log}\;Z/\partial U\partial \mu & \partial^{2}\;\text{log}\;Z/\partial U^{2} \end{array}{)}_{0} \end{array}$$ (8)

When *n*_0_ ≡ 0, *μ* is traced out of Eq (4) (i.e., *μ* → ∞) so that the partition function is independent of the chemical potential, *Z* = *Z*(*U*). As a result, Eq (8) reduces to

$$\begin{array}{c} \frac{1}{\left\langle \delta {\hat{U}}^{2}\right\rangle }=-(\frac{\partial^{2}W}{\partial U^{2}}{)}_{0}=M(\frac{\partial^{2}\;\text{log}\;Z}{\partial U^{2}}{)}_{0}=-M(\frac{\partial \langle \sigma \rangle }{\partial U}{)}_{0} \end{array}$$ (9)

The linear-response term on the right hand side of (9) is related to the variance of *σ* by the fluctuation-dissipation theorem [37]. Thus, ${\langle \delta {\hat{U}}^{2}\rangle }^{-1}=M\langle \delta \sigma^{2}\rangle$.

The model of Eq (4) readily extends to experiments with mixed sex/species, so long as distinct species can share a pot without harming one another:

$$\begin{array}{c} H\left(\vec{n}\right)=\mu \left(n_{0}^{f}+n_{0}^{m}\right)+\frac{U_{ff}}{2}\sum_{i=1}^{N}n_{i}^{f}\left(n_{i}^{f}-1\right)+\frac{U_{mm}}{2}\sum_{i=1}^{N}n_{i}^{m}\left(n_{i}^{m}-1\right)+U_{fm}\sum_{i=1}^{N}n_{i}^{f}n_{i}^{m} \end{array}$$ (10)

Here $n_{i}^{f}\left(n_{i}^{m}\right)$ is the number of females (males) occupying pot *i* out of *N* and $n_{0}^{f}\left(n_{0}^{m}\right)$ is the number of outsiders; similarly, the subscripts of *U* denote sex and take the values *m* or *f* accordingly. This model allows different interactions between sexes. For example, *U*_*fm*_ ≤ 0 ≤ *U*_*ff*_ ≤ *U*_*mm*_ would describe animals having attractive between-sex interactions and repulsive within-sex interactions, with the females being more social than the males.

The interaction parameters in Eq (10) are determined by maximum likelihood estimation. In full analogy with Eq (7)

$$\begin{array}{c} \underset{\vec{x}}{\text{max}}\;W\left(\vec{x}\right)=\underset{\vec{x}}{\text{max}}\sum_{m=1}^{M}\;\text{log}\;P\left[\vec{n}\left(m\right);\vec{x}\right] \end{array}$$ (11)

where $\vec{x}\equiv \left(\mu ,U_{ff},U_{mm},U_{fm}\right)$. The errors in estimating these parameters are given by the 4 × 4 inverse of the Hessian matrix, evaluated at the maximum likelihood solution ${\vec{x}}_{0}$ [compare to Eq (8)]:

$$\begin{array}{c} {}[\langle \delta {\hat{x}}_{i}\delta {\hat{x}}_{j}\rangle {]}^{-1}=M(\partial^{2}\;\text{log}\;Z/\partial x_{i}\partial x_{j}{)}_{0}\;,\;\left(i,j=1,2,3,4\right) \end{array}$$ (12)

As a result, the uncertainty levels of the observables $\vec{y}\equiv \left(n_{0},\sigma_{ff},\sigma_{mm},\sigma_{fm}\right)$ have two sources: one, due to the intrinsic fluctuations which occur as the animals keep moving between different occupancy configurations, and the other, due to errors in estimating the interaction parameters. Expanding the error-matrix $\langle \delta {\hat{y}}_{i}\delta {\hat{y}}_{j}\rangle$ to leading order in (1/*M*) one finds:

$$\begin{array}{c} \left\langle \delta {\hat{y}}_{i}\delta {\hat{y}}_{j}\right\rangle ={\left\langle \delta y_{i}\delta y_{j}\right\rangle }_{c}+{\left(2M\right)}^{-1}\sum_{k\ell }{\left\langle \delta y_{i}\delta y_{j}\delta y_{k}\delta y_{\ell }\right\rangle }_{c}\left\langle \delta {\hat{x}}_{k}\delta {\hat{x}}_{\ell }\right\rangle \;,\;\left(i,j,k,\ell =1,2,3,4\right) \end{array}$$ (13)

where $\langle \delta {\hat{x}}_{i}\delta {\hat{x}}_{j}\rangle$ is the error-matrix given by Eq (12) and 〈⋯〉_*c*_ are the 2 and 4-point connected correlations for a set of known parameters. The last term on the right hand side of (13) vanishes as the number of experiments *M* → ∞. The first term, however, is controlled by the size of the system (decreases as *K*, *N* → ∞, while the density *ρ* = *K*/*N* is kept finite) and, therefore, remains relatively large for small systems.

After calculating the partition function *Z* and estimating the interaction parameters and their errors [as is given in Eqs (11) and (12)], one obtains the distribution function of pot occupancies for any numbers of pots and animals (*N*, *K*) and for an arbitrary mix of sexes (*K* = *K*_*f*_ + *K*_*m*_). We follow this procedure in the Results section.

## Results and discussion

### Bucket and tank observations

We did not observe obviously aggressive interactions between *O. laqueus* individuals in our bucket field experiments, occasional color flashes because of disturbances from researchers with flashlights notwithstanding. Instead, *O. laqueus* would often sit in buckets with arms or bodies in contact with one another, and at times partially atop one another. In contrast to other species such as *Abdopus aculeatus* and *Octopus incella* that were also present in the field but encountered much less frequently, *O. laqueus* rarely attempted to escape from the open buckets, despite the potential stresses of collection, dense conditions, and limited seawater within the buckets.

Our bucket observations suggested that *O. laqueus* might thrive in communal aquaria and we explored maintaining them in shared tanks without lids in the lab. We found that for over 100 animals of mixed sexes and a range of sizes, only a few *O. laqueus* ever escaped or disappeared from open tanks housing 3-15 animals at a time. At least one incident of escape appeared to be related to poor water conditions that arose unexpectedly, while another involved a very young juvenile that was much smaller than any other *O. laqueus* brought in from the field. Three young juveniles that were shipped from Japan to the United States and raised in an open communal tank for almost five months matured and mated, with hatchlings appearing healthy and with the mother dying naturally from senescence after hatching. These results demonstrate that it is possible to collect, ship, and culture wild-caught *O. laqueus* for up to several months in open communal tanks with little risk of escape, and that the animals appear to thrive and complete their life cycle, including sexual maturation, mating, and hatching of the next generation.

As expected for a nocturnal octopus, we observed that *O. laqueus* roamed their open communal tanks at night, hunting and eating prey, and interacting with brief arm or sucker contact that did not appear to be aggressive. Each morning, octopus would select a pot to occupy for the day, rarely remaining outside all pots within the tank. Surprisingly, even in tanks with at least as many pots as octopus, multiple individuals would share a single pot for the day, often within arm’s reach or in non-aggressive contact with one another inside the pot. As in our bucket experiments, these observations of non-aggressive co-occupancy of a communal tank over periods of days to months suggest that *O. laqueus* is much more socially tolerant then we expected based on studies of other octopus species, where octopus are housed in isolation or must be size-matched and well fed. That two or more *O. laqueus* will share not only a tank but even a pot serving as a den is remarkable, as den sharing in aquaria or in the field was until now unreported for octopus, so far as we know, outside the exceptional occurrence of mate-pair bonding in *Octopus* LPSO, wherein a mating male and female will share a den for several days [14].

### Replicates and balance in the experimental design

An advantage of laboratory experiments on behavior over those done in the field is that the degree of control often allows the number of replicates and balance in treatments to match the ideal experimental design. However, because animals could be obtained only with considerable difficulty, our control was limited. In this context, the experiments above have a relatively low number of replicates (2 for each of the treatments) and are at times unbalanced with respect to sex or size. Specifically, there are only two replicates of each of the FF, MM, and FM Pots Equal (PE) treatments and of the Pots Limited treatment (PL). Further, size and sex representations are not balanced across a given treatment to a varying degrees for all treatments. The low number of replicates and unbalance in the data versus an ideally balanced experimental design is due largely to limitations encountered in collecting *O. laqueus* within a limited window to do experiments. Importantly, despite these limitations, we are able to identify with statistical significance that *O. laqueus* share dens yet they are far from being neutral independent animals.

### Observations of pots occupancy

Daily pots occupancies, for both Pots Equal and Pots Limited experiments and observed over 45 days, are shown in Tables 1–4. Each table specifies the number of available pots *N*, the number of females *K*_*f*_ or males *K*_*m*_ in the tank (*K*_*m*_ + *K*_*f*_ = *K*), the number of animals that were found each day inside pot *i*, *n*_*i*_ (*i* = 1, …, *N*), and the number animals that stayed in the open space outside the clay pots, *n*_0_, The tables also specify the daily sharing levels *S*_*d*_ [Eq (1)] and the pairwise linkage, i.e., the total number of pairs formed by female-female, male-male and female-male, respectively:

$$\begin{array}{c} \sigma_{ff}=\frac{1}{2}\sum_{i=1}^{N}n_{i}^{f}\left(n_{i}^{f}-1\right)\;,\sigma_{mm}=\frac{1}{2}\sum_{i=1}^{N}n_{i}^{m}\left(n_{i}^{m}-1\right)\;,\sigma_{fm}=\sum_{i=1}^{N}n_{i}^{f}n_{i}^{m} \end{array}$$ (14)

As opposed to *S*_*d*_, the pairwise linkage is sensitive both to the sex and to the density of animals in a pot.

#### Occupancy in Pots Equal experiments

For Pots Equal experiments, pot occupancy across 31 days of observation having an equal number of octopus and pots (*N* = *K*) ranged from zero to three animals in a pot. The total number of sharing animals per day, *S*_*d*_, ranged from zero to five. Specifically, we found that *S*_*d*_ ≥ 2 i.e., at least two animals were sharing a pot, in 19 out of 31 days (61%). Then, looking at the subset of *N* = *K* = 6 (omitting the all-male *N* = 5 replicates in Table 4a, and a single incident with *n*_0_ ≠ 0 in Table 1b), we found that *S*_*d*_ ≥ 2 in 16 out of 25 days (64%). These numbers are sufficiently high to demonstrate that *O. laqueus* are not totally solitary and can be tolerant of sharing a clay pot or den with one or more individuals. At the same time, it’s clear that the animals are far from being neutral (indifferent to the presence of others) because, for *K* independent animals distributed among *N* jars, the probability of non-sharing would be [see Eq (2)] *P*(*S*_*d*_ = 0) = *K*!*N*^−*k*^ = 1.5%. Averaging the occupation numbers of all the pots over 25 days, we also found that

$$\begin{array}{c} {\bar{n}}_{1}=1.04,{\bar{n}}_{2}=1.16,{\bar{n}}_{3}=0.96,{\bar{n}}_{4}=0.76,{\bar{n}}_{5}=1.08,{\bar{n}}_{6}=1.00 \end{array}$$ (15)

verifying thereby that all pots are statistically identical (the deviations compared to the expected mean values of ${\bar{n}}_{i}=1$ were tested using a 6-level 1-way-ANOVA and turned out to be insignificant with a p-value = 0.23). Thus, clay pot or den selection is not an entirely random process, and there is an anti-social behavioral component at play, keeping pot sharing at levels lower than predicted by a neutral random model.

#### Sex analysis

Sex analysis of the pot occupancy data suggests that the anti-social behavior component is coming primarily from male-male interactions but occurs at statistically significant levels even in all-female tanks. Indeed, the average sharing number of all-females configurations is ${\bar{S}}_{d}^{f}=2.7$ whereas the all-males average sharing is ${\bar{S}}_{d}^{m}=\left(0.8+0.4\right)/2=0.6$ [for estimation of the error-bars, see Eqs (20a) and (20b)]. Therefore, females are much friendlier than males, however, both sexes are less friendly than neutral animals. For comparison, the average sharing levels of independent animals are:

$$\begin{array}{c} S_{0}=\{\begin{array}{cccc} \frac{4651}{1296} & = & 3.59, & \text{for}\;\;N=K=6\\ \frac{369}{125} & = & 2.95, & \text{for}\;\;N=K=5 \end{array} \end{array}$$ (16)

Furthermore, considering the case of mixed sexes (Table 1), one can verify that most of the sharing events (8/10) were by female-male pairs. This tendency persists even in the case of limited number of dens, *N* < *K* (Table 2), so that in all experiments ${\bar{\sigma }}_{fm}>{\bar{\sigma }}_{ff}$.

#### Occupancy in Pots Limited experiments

For the Pots Limited experiments, pot occupancies across all the experiments having more octopus than pots (*N* < *K*) ranged from two to four animals in a pot (Table 2), and the daily sharing numbers ranged accordingly from four to six sharing animals per day. We found that, out of fourteen tank examinations, *S*_*d*_ = 4, 6 each occurred twice and *S*_*d*_ = 5 occurred 10 times (71%). However, limiting dens also increased the number of ‘outsiders’(namely those animals, either females or males, that stay in the open environment outside the pots) so that a solitary octopus was found outside the pots on most days. Specifically, we found that *n*_0_ = 0 occurred only twice (14.3%), *n*_0_ = 1 occurred 11 times (78.6%), and *n*_0_ = 2 happened once (7.1%). Thus, overall, limiting dens increased the amount of social sharing but, at the same time, forced some fraction of the animals to stay out of the dens.

### Tests against neutrality

The sample-mean and sample-variance of *S*_*d*_ for all the experimental treatments are shown in Table 6. Also shown the mean values of the neutral model, that serves as a null hypothesis, and the corresponding p-values of the one-sample t-tests. The deviations from neutrality are significant for all experimental setups, except FF (all females) with a p-value = 0.07. Obviously, the significance is further increased by pooling two replicates together. As is readily verified by looking at the main lobe of the temporal correlation function $C(\tau )=\bar{\delta S_{d}\left(m\right)\delta S_{d}\left(m-\tau \right)}$ with $\delta S_{d}\equiv S_{d}-{\bar{S}}_{d}$, the correlation time is less than a day in all the experiments. Furthermore, the power ratio of main-lobe to the side-lobes of *C*(*τ*) is 8dB and the relaxation time [38] is $\tau_{0}=2\int_{0}^{\infty }d\tau C\left(\tau \right)=0.82$ days. Thus, practically, the daily measurements are statistically independent.

**Table 6:** The sharing levels compared to the neutral model for eight experimental setups and their combinations. ${\bar{S}}_{d}$ is the sample mean, Σ_*d*_ the sample standard deviation and *S*_0_ is the neutral reference mean.

Exp.	Tab.	Tank	# days	# pots	Fem	Male	Mean	Std	Ref.	p-val
			*M*	*N*	*K*_*f*_	*K*_*m*_	${\bar{S}}_{d}$	Σ_*d*_	*S*_0_	
1	1a	T3	5	6	6	0	3.2	1.1	3.59	0.2360
2	1b	T4	5	6	6	0	2.2	2.3	3.59	0.1225
1+2	(FF)		10	6	6	0	2.7	1.67	3.59	0.0731
3	2a	T1	5	5	0	5	0.8	1.1	2.95	0.0059
4	2b	T2	5	6	0	6	0.4	0.9	3.59	0.0007
3+4	(MM)		10	5,6	0	5,6	0.6	1.01	3.59	10^−5^
5	3a	T1	6	6	3	3	1.0	1.1	3.59	0.0011
6	3b	T3	5	6	3	3	2.8	1.1	3.59	0.0914
5+6	(FM)		11	6	3	3	1.8	1.40	3.59	0.0009
7	4a	T2	7	2	3	3	5.1	0.7	5.71	0.0212
8	4b	T4	7	2	4	2	4.9	0.4	5.71	0.0003
7+8	(PL)		14	2	3,4	3,2	5.0	0.55	5.71	0.0001

### The effect of treatments

To test for significant fixed effects among daily sharing levels *S*_*d*_, we performed a 4-level-1-way unbalanced ANOVA test using Matlab^©^. A 2-way-ANOVA is not applicable in our case, due to insufficient degrees of freedom (dof) which leads to singular cross terms (we only have 3 dof at our disposal, whereas a full 2-way analysis would require 5 dof). The ANOVA levels correspond to the above mentioned 4 groups: MM, FF, FM, and PL. As shown in Table 7, the p-value is extremely low, of the order $O(10^{-9})$. This allows us to proceed in trying to identify the significant treatments among the 4 groups using six post-hoc t-tests. To account for varying sample sizes, we used the Welch unbalanced two-sample t-tests. As the mean of the mixed FM group lies in between the means of all-females and all-males groups, the differences |FF − FM| and |MM − FM| are insignificant. Other contrasts, including the |FM − PL| between mix sexes at different densities, are statistically significant (see Table 7). These contrasts remain significant after correcting by a factor of 12 (a factor of 6 is the Bonferroni correction and another factor of 2 comes from doing double-sided tests).

**Table 7:** Effect of treatments {FF,MM,FM,PL} (a) ANOVA table (b) post-hoc t-tests (before multiple-test corrections).

(a)	Ssq	dof	Ssq/dof	F-val	p-val	(b)	MM	FM	PL
Group	123.3	3	41.1	26.9	8.8 × 10^−10^	FF	0.0042	0.1131	0.0017
Error	62.6	41	1.53			MM		0.0261	1 × 10^−7^
Total	185.9	44				FM			1 × 10^−5^

### Estimation of the maximum entropy interaction parameters

The *S*_*d*_ statistic is not sensitive to sex-mixtures and local densities and therefore cannot resolve, for example, the difference between the two replicates of Table 2. A possible way to overcome such limitations, as well as un-balanced and un-factorised experimental designs, is by using the max-ent model. As explained in the Methods section, an estimation of the interaction parameters $\vec{x}=\left(\mu ,U_{ff},U_{mm},U_{fm}\right)$ involves a computation of the partition function $Z(\vec{x})$, and depends on the empirical data $\vec{y}=\left({\bar{n}}_{0},{\bar{\sigma }}_{ff},{\bar{\sigma }}_{mm},{\bar{\sigma }}_{fm}\right)$ given in Table 8.

**Table 8:** Summary of measurements for eight experimental setups and combinations, showing the average number of outsiders ${\bar{n}}_{0}$ (females and males) and the average number of links $\left({\bar{\sigma }}_{ff},{\bar{\sigma }}_{mm},{\bar{\sigma }}_{fm}\right)$.

Exp.	Tab.	Tank	# days	# pots	Fem	Male	# config	Outsiders		#links		
			*M*	*N*	*K*_*f*_	*K*_*m*_	Ω	${\bar{n}}_{0}^{f}$	${\bar{n}}_{0}^{m}$	${\bar{\sigma }}_{ff}$	${\bar{\sigma }}_{mm}$	${\bar{\sigma }}_{fm}$
1	1a	T3	5	6	6	0	462	0	-	1.60	-	-
2	1b	T4	5	6	6	0	462	0	-	1.40	-	-
1+2	(FF)		10	6	6	0	462	0	-	1.50	-	-
3	2a	T1	5	5	0	5	126	-	0	-	0.40	-
4	2b	T2	5	6	0	6	462	-	0	-	0.20	-
3+4	(MM)		5+5	5,6	0	5,6	588	-	0	-	0.30	-
5	3a	T1	6	6	3	3	1120	0	0	0.17	0	0.33
6	3b	T3	5	6	3	3	1680	0.20	0	0.20	0	1.20
5+6	(FM)		11	6	3	3	1680	0.09	0	0.18	0	0.73
7	4a	T2	7	2	3	3	100	0.71	0.14	0.43	0.86	3.29
8	4b	T4	7	2	4	2	90	0.71	0.29	1.29	0.14	2.86
7+8	(PL)		7+7	2	3,4	3,2	190	0.71	0.21	0.86	0.50	3.08

#### Interaction parameters of single-sex populations

Let’s start with the simpler estimation for a single-sex populations, described by Eq (4). In this case, the total number of configurations for *K* identical animals occupying *N* pots is

**Fig 3:**
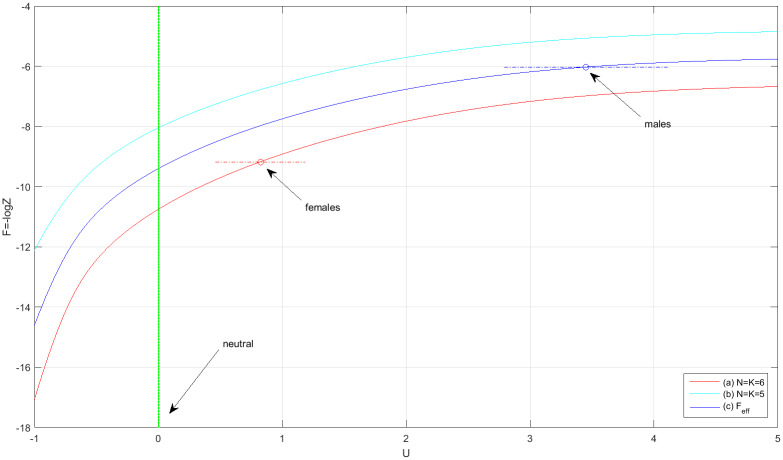
The free energy, *F* = −log *Z*, as a function of the interaction parameter *U*. (a) for *N* = *K* = 6 (b) for *N* = *K* = 5. (c) The combined free-energy $F_{\text{eff}}=\frac{1}{2}\left(F_{1}+F_{2}\right)$. The estimated values of the interaction, *U*_*ff*_ and *U*_*mm*_ [Eq (18)], are shown together with their corresponding error-bars. Both females and males are far from being neutral (green line, *U* = 0). In a single-sex environment females are more social than males.

Ω=(N+K-δK) (17)

where *δ* ≡ 1 if ${\bar{n}}_{0}=0$ and zero otherwise (${\bar{n}}_{0}\neq 0$ means that the animals can dwell somewhere in the tank outside the pots. Combinatorially, this amounts to having an additional available ‘slot’). Referring to the first two rows in Table 8, with *N* = *K* = 6 and *δ* = 1, the number of configurations is Ω = (2*N* − 1)!/[*N*!(*N* − 1)!] = 462. The calculation of the partition function is, therefore, amendable to numerical computation. The partition function (more precisely, the free energy *F* ≡ −log *Z*) as a function of *U* is plotted in Fig 3. Therefore, with *F*(*U*) given, and by solving Eq (6) $\bar{\sigma }=\partial F/\partial U$ for *U*, we find that

$$\begin{array}{c} U_{ff}=0.82\pm 0.36\;,\;U_{mm}=3.45\pm 0.66 \end{array}$$ (18)

As expected, in a non-mixed environment females are friendlier than males. However, compared to neutral animals (*U* = 0) both sexes exhibit significant repulsive interaction. The *t*-statistic for the difference between sexes is $t=|3.45-0.82|/\sqrt{0.36^{2}+0.66^{2}}=3.50$ with a *p*-value = 0.002. These results are based on combining two replicates consisting of a total of 10 measurements for each sex (see Tables 3 and 4). For males, since *N*_1_ = *K*_1_ = 5 and *N*_2_ = *K*_2_ = 6, the combined free energy for two replicates is given by a weighted average,

$$\begin{array}{c} F_{\text{eff}}=\left(M_{1}F_{1}+M_{2}F_{2}\right)/\left(M_{1}+M_{2}\right) \end{array}$$ (19)

and Eq (6) takes the form: ${\bar{\sigma }}_{\text{eff}}=\partial F_{\text{eff}}/\partial U$, where ${\bar{\sigma }}_{\text{eff}}=\left(M_{1}\bar{\sigma_{1}}+M_{2}{\bar{\sigma }}_{2}\right)/\left(M_{1}+M_{2}\right)$ is the effective number of links. Eq (19) demonstrates how independent data sets (in this example, unbalanced male replicates 1&2) are compiled together into a single set with proper averaging.

**Fig 4:**
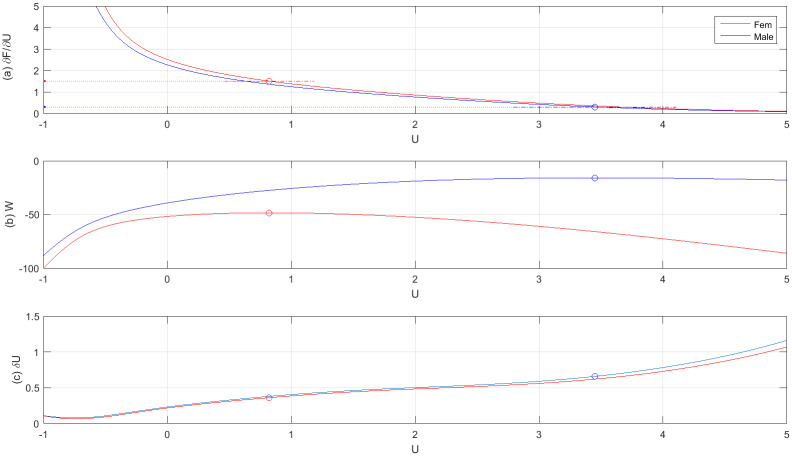
(a) The average number of links 〈*σ*〉 = ∂*F*/∂*U* and (b) the log-likelihood function *W* = *F* − *U*〈*σ*〉 showing that *W* assumes its maximal value when $\langle \sigma \rangle =\bar{\sigma }$. (c) the fluctuation $\delta \hat{U}$. (solid-red line: females, solid-blue line: males).

We computed the following quantities as a function of the interaction parameter *U* (Fig 4): (i) the average number of links 〈*σ*〉 = ∂*F*/∂*U* (ii) the canonical distribution $P\left(\vec{n}\right)=g\left(\vec{n}\right)\text{exp}\left[-H\left(\vec{n}\right)\right]/Z\left(U\right)$ and the log-likelihood function $W\equiv \sum_{m=1}^{M}\;\text{log}\;P\left({\vec{n}}_{m}\right)=M(F-U\bar{\sigma })$ and (iii) the fluctuation $\delta \hat{U}$ according to Eq (9). As a consistency check of the model, we also calculated the average sharing-number in terms of the canonical distribution $P(\vec{n})$. Namely, $\langle S_{d}\rangle =\sum_{\vec{n}}P\left(\vec{n}\right)\delta \left[S_{d}-\sum_{i}n_{i}I\left(n_{i}-2\right)\right]$. Note that, by its construction, the canonical distribution $P(\vec{n})$ always reproduces the average number of links $\langle \sigma \rangle =\bar{\sigma }$. However, functions like *P*_obs_(*S*_d_) and *P*_cal_(*S*_d_), i.e., the empirical and derived distributions of *S*_*d*_, are more complicated objects and, as such, they don’t necessarily need to agree with each other [consistency is nevertheless maintained, because *D*[*P*_obs_(*S*_d_)||*P*_cal_(*S*_d_)] is minimized exactly at the same value of *U* which solves the maximum likelihood condition $\bar{\sigma }=\partial F/\partial U$]. We found that the average sharing numbers, calculated at the corresponding maximum likelihood solutions (18) (i.e., *U*_*ff*_ = 0.82, *U*_*mm*_ = 3.45), are

$$\begin{array}{c} \left\langle S_{d}^{f}\right\rangle =2.72\pm 0.35\;,\;\left\langle S_{d}^{m}\right\rangle =0.60\pm 0.30 \end{array}$$ (20a)

These values are in good agreement with the experimental results,

$$\begin{array}{c} {\bar{S}}_{d}^{f}=2.70\pm 0.56\;,\;{\bar{S}}_{d}^{m}=0.6\pm 0.44 \end{array}$$ (20b)

**Fig 5:**
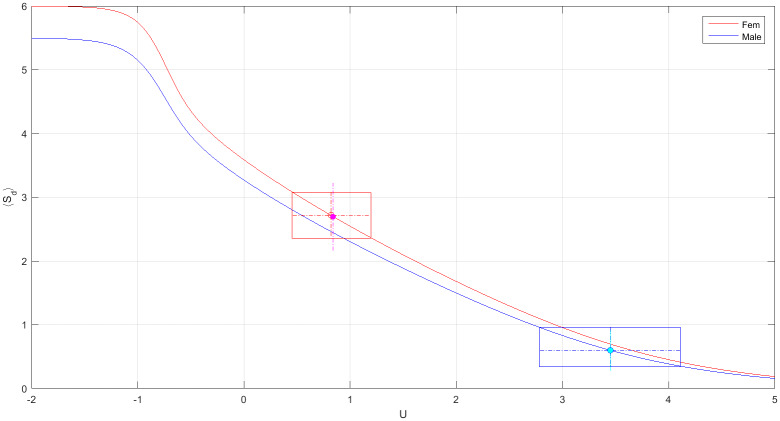
The average sharing number 〈*S*_*d*_〉 as a function of *U* for females (red) and males (blue). Females: 〈*S*_*d*_〉 evaluated at *U*_*ff*_ = 0.82 gives $\langle S_{d}^{f}\rangle =2.72\pm 0.35$ (indicated by the red-rectangle) and the experimental value is ${\bar{S}}_{d}^{f}=2.7\pm 0.56$ (dotted-magenta). Males: 〈*S*_*d*_〉 evaluated at *U*_*mm*_ = 3.45 gives $\langle S_{d}^{m}\rangle =0.60\pm 0.30$ (blue-rectangle) and the experimental value is ${\bar{S}}_{d}^{m}=0.6\pm 0.44$ (dotted-cyan).

In particular, the estimated errors in Eq (20a) are smaller than the empirical ones and, as shown in Fig 5, the empirical values lay well within the estimated confidence levels. Since, ${\bar{S}}_{d}\left(U=3\right)≃1$ (Fig 5), it follows that, for *U* ≤ 3 one typically observes at least one pot with sharing animals, whereas for *U* > 3 sharing is much suppressed. Also note that both values in (20a) differ significantly from the expected sharing level of neutral animals, Eq (16).

**Fig 6:**
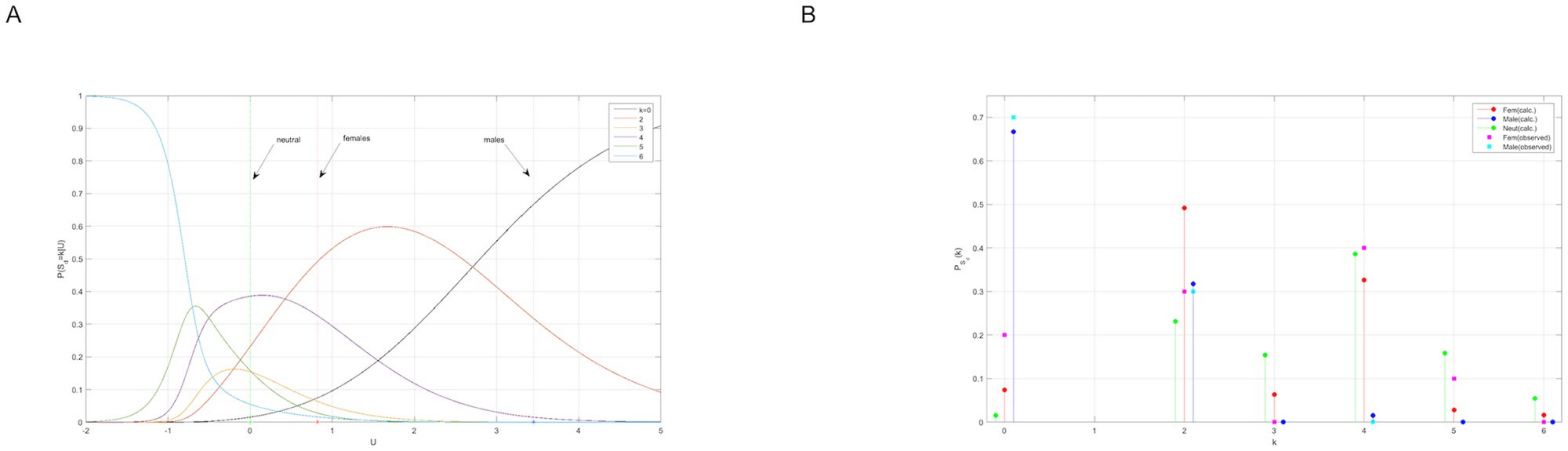
The sharing distribution as a function of the interaction parameter, $P_{S_{d}}\left(k|U\right)$ {*k* = 0, 2, …, *K*} for *K* = 6 animals in *N* = 6 jars. (a) The probability of non-sharing (black solid line) is 7% for females (red dot at *U* = 0.82) and for males 67% (blue dot at *U* = 3.45). For neutral animals the probability of non-sharing is 1.5% (green dot at *U* = 0). (b) Comparison of the sharing probability for females (red), males (blue) and neutral animals (green). The empirical probability, obtained by averaging of 10 days, is also shown for females (magenta) and males (cyan).

**Fig 7:**
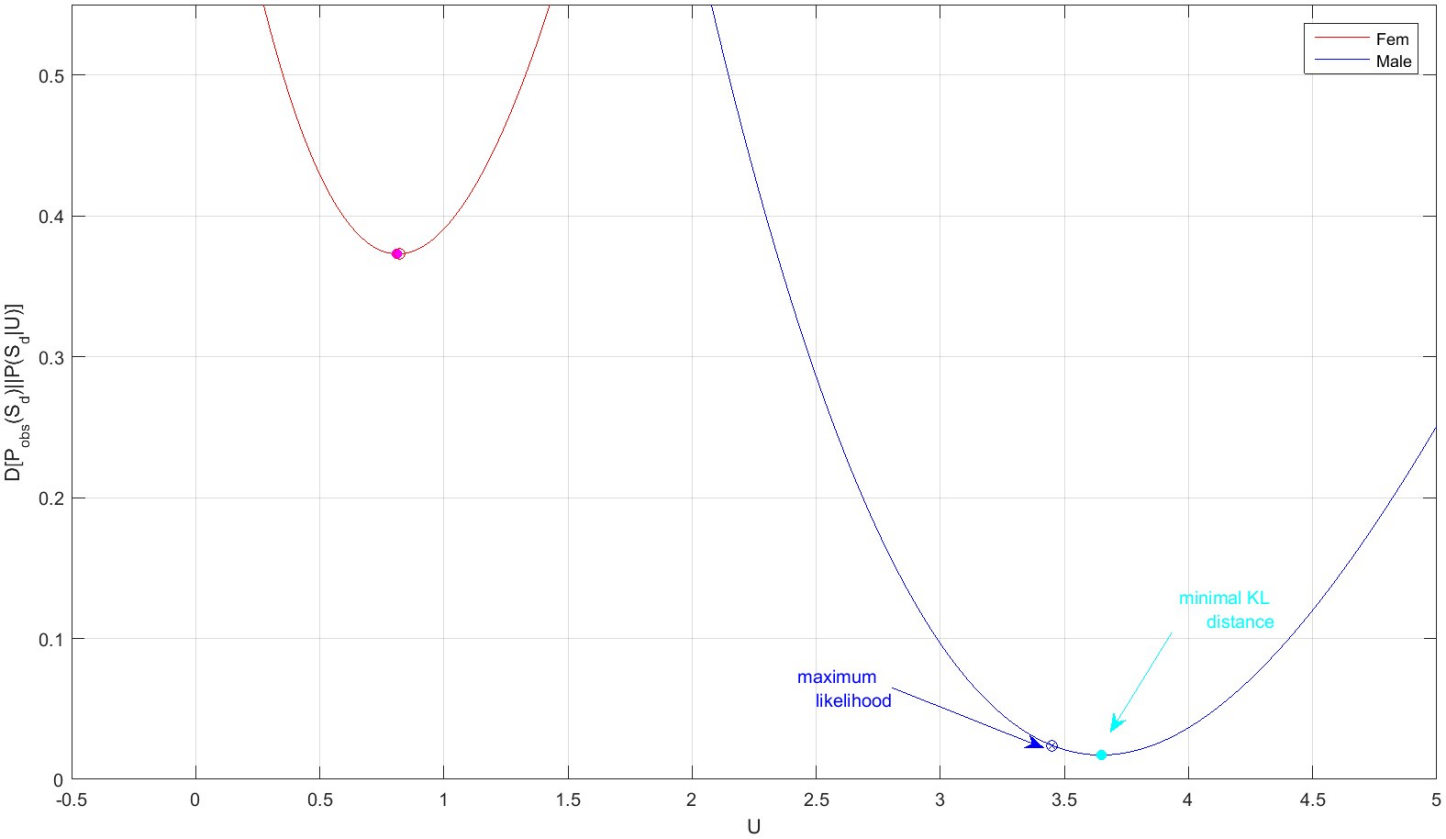
(a) The KL-distance *D*[*P*_obs_(*S*_d_)||*P*(*S*_d_|*U*)], between the empirical sharing distribution and the calculated sharing distribution as a function of *U*, for females (red) and males (blue).

**Fig 8:**
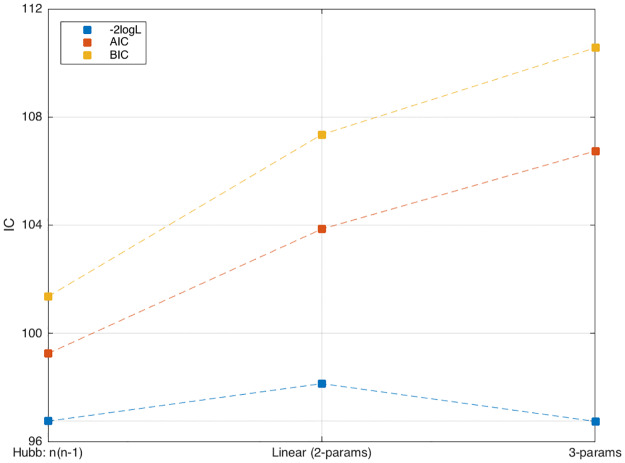
Comparison of AIC (Akaike Information Criterion) for 3 polynomial models with *K* = 6 females in *N* = 6 pots. (a) Hubbard: $H_{1}\left(\vec{n}\right)=\left(U/2\right)\sum_{i}n_{i}\left(n_{i}-1\right)$. (b) linear $H_{2}\left(\vec{n}\right)=\left(V/2\right)\sum_{i}n_{i}I\left[n_{i}-2\right]$. (c) 3-parameters: $H_{3}\left(\vec{n}\right)=\sum_{i}W_{i}\left(n_{i}\right)$, with *W*_*i*_(0) = 0, *W*_*i*_(1) = *ω*_1_, *W*_*i*_(2) = *ω*_2_, *W*_*i*_(*n*_*i*_ ≥ 3) = *ω*_3_. The maximum likelihood *L* is obtained, respectively, for *U* = 0.82, *V* = 0.52 and *ω* = (0.78, 2.41, 4.74). Here AIC = −2 log *L* + 2*p* + 2*p*(*p* + 1)/(*M* − *p* − 1), BIC = −2 log *L* + 2*p* log *M*, where *p* = (1, 2, 3) is the number of parameters and *M* = 10 is the numbers of measurements.

The full sharing distribution as a function of the interaction parameter, $P_{S_{d}}\left(k|U\right)$ {*k* = 0, 2, …, *K*}, is shown in Fig 6 (for *N* = *K* = 6). We find that the non-sharing probability $P_{0}\equiv P_{S_{d}}\left(k=0|U\right)$, evaluated at the maximum likelihood points Eq (18), is $P_{0}^{f}=7\%$ for females and $P_{0}^{m}=67\%$ for males. Clearly, both values are larger than the non-sharing probability of neutral animals. More generally, we examined the Kullback-Leibler distance between the empirical sharing distribution, *P*_obs_(*k*) = *M*^−1^ ∑_*m*_ δ[*S*_*d*_(*m*) − *k*], and the probability $P_{S_{d}}\left(k|U\right)$ calculated as a function of *U* by using the distribution function $P(\vec{n})$. We found (Fig 7), that the KL-distance *D*[*P*_obs_(*S*_d_)||*P*_cal_(*S*_d_|*U*)] assumes its minimal value—respectively for females and males, at *U* = (0.82, 3.45) which is again very close to the maximum likelihood solution Eq (18). Remarkably, this holds even though the number of observations, *M* = 10, is pretty small. In addition, the one-parameter model $H_{1}\left(\vec{n}\right)=\left(U/2\right)\sum_{i}n_{i}\left(n_{i}-1\right)$, resulting from Eq (4) by setting *μ* → ∞, has the smaller AIC as compared other polynomial models (see Table 9 as well as Fig 8).

**Table 9:** Comparison of AIC (Akaike Information Criterion) for 3 polynomial models with K = 6 females in N = 6 pots, measured over M = 10 days. *AIC* = −2 log *L* + 2*p* + 2*p*(*p* + 1)/(*M* − *p* − 1), BIC = −2 log *L* + 2*p* log *M*, where *p* = (1, 2, 3) is the number of parameters.

Model		parameters	loglike	AIC	BIC
Hubbard	$H_{1}\left(\vec{n}\right)=\left(U/2\right)\sum_{i}n_{i}\left(n_{i}-1\right)$	*U* = 0.82	-48.3756	99.2512	101.3564
Linear	$H_{2}\left(\vec{n}\right)=\left(V/2\right)\sum_{i}n_{i}I\left[n_{i}-\nu \right]$	*V* = 0.52, *ν* = 2	-49.0697	103.8537	107.3497
3rd order	$H_{3}\left(\vec{n}\right)=\left(1/2\right)\sum_{i}W_{i}\left(n_{i}\right)$	*W*(0) = 0, *W*(1) = 0.78, *W*(2) = 2.41, *W*(*n* ≥ 3) = 4.74	-48.3707	106.7413	110.5569

#### Interaction parameters of mixed populations

The total number of configurations associated with mixed populations as in Eq (10) is

Ω=Ωf×Ωm=(N+Kf-δfKf)(N+Km-δmKm) (21)

where $K_{f}=\sum_{i=0}^{N}n_{i}^{f}\;\left(K_{m}=\sum_{i=0}^{N}n_{i}^{m}\right)$ is number of females (males) and $\delta_{f}\equiv \delta \left({\bar{n}}_{0}^{f}\right)$ [*δ*_*f*_ = 1, if ${\bar{n}}_{0}^{f}=0$; *δ*_*f*_ = 0 otherwise; and similarly for $\delta_{m}\equiv \delta \left({\bar{n}}_{0}^{m}\right)$].

We first applied the extended model of Eq (10) to the case of mixed sexes with equal number of animals and pots: *K*_*f*_ = *K*_*m*_ = 3, *N* = *K*_*f*_ + *K*_*m*_ = 6. Referring to Table 8, we find that *δ*_*f*_ = 0 and *δ*_*m*_ = 1 (${\bar{n}}_{0}^{f}=1/11$, ${\bar{n}}_{0}^{m}=0$). The number of configurations is then Ω=(93)(83)=4704. Combining tanks #1 and #3, we also observed that ${\bar{\sigma }}_{mm}=0$ (males never shared pots with any other males for 11 days). Therefore, tracing out *U*_*mm*_ and solving ∂*F*/∂*μ* = 1/11, ∂*F*/∂*U*_*ff*_ = 2/11, ∂*F*/∂*U*_*fm*_ = 8/11, we find that

$$\begin{array}{c} \mu =2.59\pm 1.04\;,U_{ff}=1.64\pm 0.81\;,\;U_{fm}=1.49\pm 0.48 \end{array}$$ (22)

The female-female interaction is consistent with the previous result [Eq (18)] obtained in a single-sex environment. The chemical potential *μ* being of the same order of magnitude as *U*_*ff*_ is sufficient to prevent females from staying outside the pots. The female-male interaction *U*_*fm*_ is much less repulsive than either *U*_*ff*_ or *U*_*mm*_. The error estimates in (22) are obtained, as in (12), by calculating the Gaussian fluctuation of the free-energy at the maximum-likelihood solution.

Next, we considered the case of dense pots *N* = 2 < *K*_*f*_ + *K*_*m*_ = 6 For tank #4, containing 4 females and 2 males that are sharing 2 pots, Eq (21) gives Ω=(64)(42)=15×6=90 configurations. For tank #2, with 3 females and 3 males Ω=(53)2=100. Such small number of configurations enables one to obtain the exact partition function and infer the four coupling constants of $H(\vec{n})$. In practice however, the number of samples *M* = 7 is also very small, so the expected accuracy of these parameters is rather low. The results are summarized in Table 10. Tank #4 looks promising: females are as social as males and the f-m interaction is on the verge of attraction

$$\begin{array}{c} \mu =2.55\pm 1.42\;,U_{ff}=1.61\pm 0.85\;,\;U_{mm}=1.61\pm 1.10\;,\;U_{fm}=0.24\pm 0.83 \end{array}$$ (23)

On the other hand, in tank #2 the males look more social than females:

$$\begin{array}{c} \mu =2.87\pm 1.55\;,U_{ff}=3.15\pm 1.25\;,\;U_{mm}=1.35\pm 1.05\;,\;U_{fm}=-0.01\pm 0.94 \end{array}$$ (24)

This ‘anomaly’ can be traced back to a high degree of individual variety (it turns out, see S4 Table, that a certain large female, named *2RG*, sits most of the time out of the pots and seems to be extremely anti-social).

**Table 10:** The estimated interaction parameters for eight experimental setups and their combinations.

Exp.	Tab.	Tank	# days	# pots	Fem	Male	ch. potent	interaction		
			*M*	*N*	*K*_*f*_	*K*_*m*_	*μ*	*U*_*ff*_	*U*_*mm*_	*U*_*fm*_
1	1a	T3	5	6	6	0	-	0.70 ± 0.49	-	-
2	1b	T4	5	6	6	0	-	0.96 ± 0.54	-	-
1+2	(FF)		10	6	6	0	-	0.82 ± 0.36	-	-
3	2a	T1	5	5	0	5	-	-	2.83 ± 0.85	-
4	2b	T2	5	6	0	6	-	-	4.15 ± 1.09	-
3+4	(MM)		5+5	5,6	0	5,6	-	-	3.45 ± 0.66	-
5	3a	T1	6	6	3	3	-	2.35 ± 1.12	-	2.83 ± 0.82
6	3b	T3	5	6	3	3	1.23 ± 1.09	0.99 ± 1.10	-	0.36 ± 0.61
(a) 5+6	(FM)		11	6	3	3	2.59 ± 1.04	1.64 ± 0.81	-	1.49 ± 0.48
(b) 7	4a	T2	7	2	3	3	2.87 ± 1.55	3.15 ± 1.25	1.35 ± 1.05	−0.01 ± 0.94
(c) 8	4b	T4	7	2	4	2	2.55 ± 1.42	1.61 ± 0.85	1.61 ± 1.10	0.24 ± 0.83
7+8	(PL)		7+7	2	3,4	3,2	2.90 ± 1.03	2.16 ± 0.72	1.33 ± 0.67	0.28 ± 0.58
**All combined**			45				3.83 ± 0.52	1.56 ± 0.31	2.61 ± 0.42	1.05 ± 0.31

#### The combined interaction parameters

All the experimental results can be treated on the same footing by combining the interaction parameters, obtained separately under different experimental conditions, into a single set of properly weighed parameters, as is done in Eq (19). Referring to Table 10 and combining together the results of five different setups, (1+2), (3+4), (5+6), (7) and (8) [see also Eqs (18), (22), (23) and (24)], we find:

$$\begin{array}{c} \mu =3.83\pm 0.52\;,U_{ff}=1.56\pm 0.31\;,\;U_{mm}=2.61\pm 0.42\;,\;U_{fm}=1.05\pm 0.31 \end{array}$$ (25)

The *t*-statistic for the difference between sexes in Eq (25) is *t* = 2.01. This contrast is lower than the corresponding single-sex statistic [Eq (18)]. However, it’s still significant with a *p*-value = 0.025.

Eq (25) specifies the most probable set of interaction parameters that are consistent with the total of 45 available measurements. These values can be used in 2 ways: first, for identifying potential outliers and second, for the prediction of the behavior over a large set of experimental designs. As an example, let’s consider *K* = 6 animals distributed among a varying number of pots *N* = (1, 2, …, 8) with several possible mixtures of sexes, *K*_*f*_ = 0, 1, …, 6 (*K*_*m*_ = *K* − *K*_*f*_). In this case, all quantities of interest, such as the number of outsiders *n*_0_ or the female-male linkage *σ*_*fm*_ (which may well affect factors like potential mating, rate of cannibalism etc.), are determined by two parameters: the specific volume *N*/*K* and the sex mixture *K*_*f*_/*K*.

**Fig 9:**
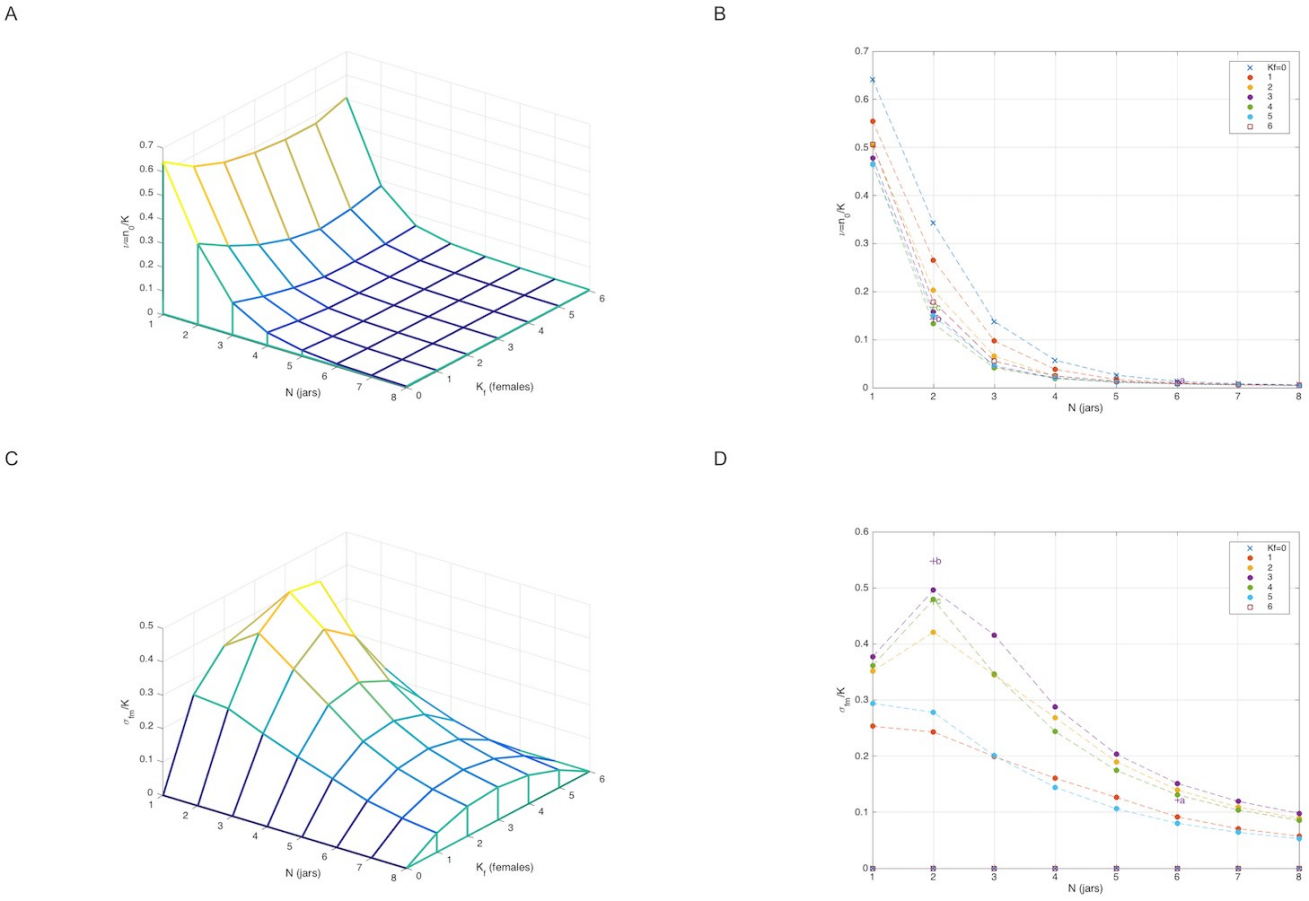
(a) The average number of outsiders as a function of (*N*, *K*_*f*_). 〈*n*_0_〉 is minimal for balanced sexes, *K*_*f*_ = *K*_*m*_. The empirical points (*b*, *c*) are close to their corresponding calculated curves. (b) The average female-male linkage 〈*σ*_*fm*_〉 as a function of (*N*, *K*_*f*_). 〈*σ*_*fm*_〉 is maximal for balanced sexes *K*_*f*_ = *K*_*m*_ and *N* = 2. The empirical points (*b*, *c*) are again close to the calculated curve however, point (*a*) looks like an outlier.

**Fig 10:**
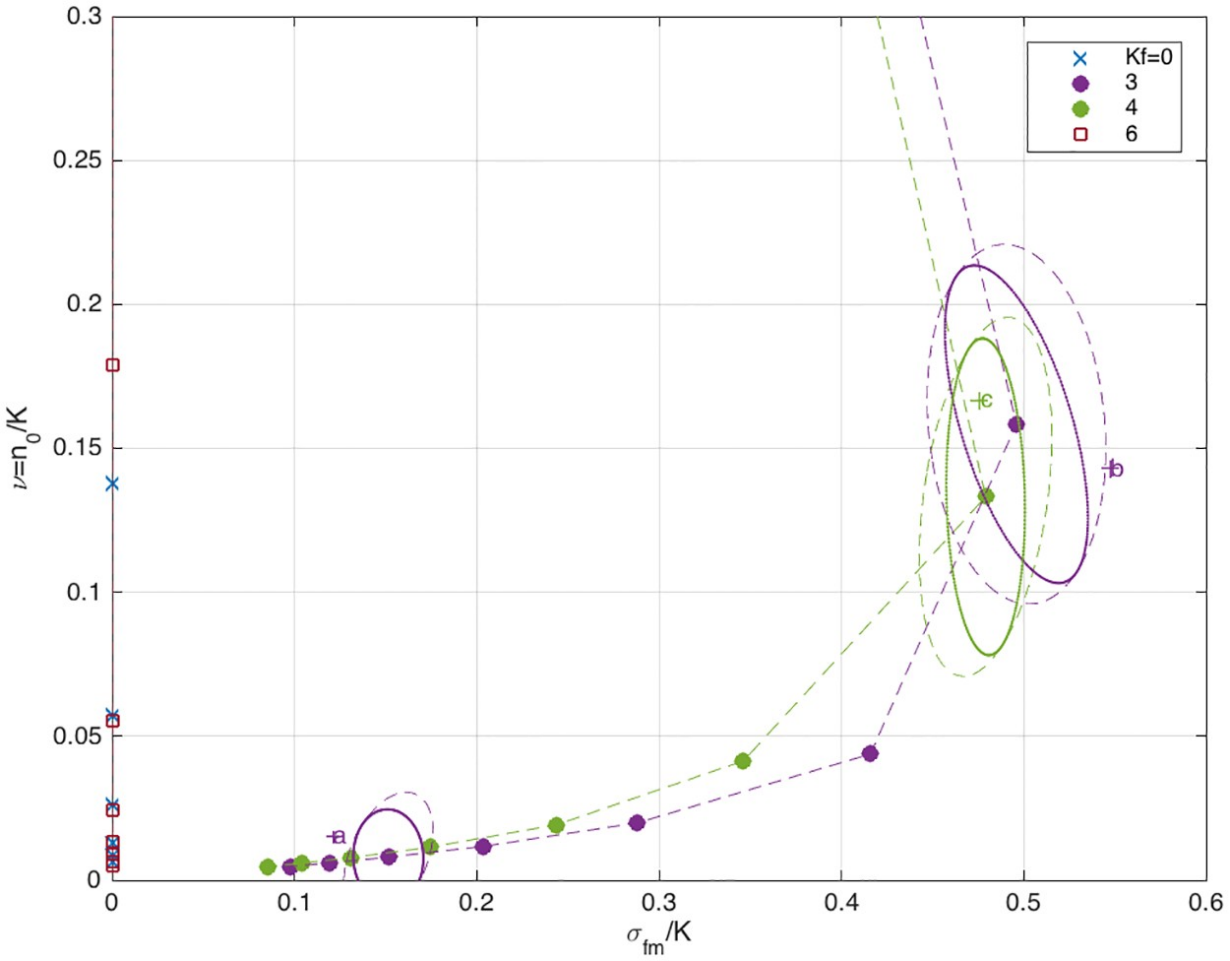
The ‘equation-of-state’ in the 〈*σ*_*fm*_〉 − 〈*n*_0_〉 plane, showing the trade-off between high female-male linkage and large number of outsiders. The ellipses of 10% uncertainty demonstrate that empirical point (*c*) lays well within the 10% error, point is (*b*) is a range of less than 11% error, and point (*a*) is more than 15% away [ellipse solid-line: finite-size 10% uncertainty for a given set of interaction-parameters, dashed-line: error in parameter estimation is included according to Eq (13)].

In Fig 9a and 9b, 〈*n*_0_〉, 〈*σ*_*fm*_〉 are shown as functions of *N* and *K*_*f*_. As expected, both 〈*n*_0_〉 and 〈*σ*_*fm*_〉 assume their maximal values when the number of pots is limited (*N* = 2) and the mixture of sexes is balanced (*K*_*f*_ = *K*_*m*_). Fig 9 suggests that the two empirical points (*b*, *c*), described by Eqs (23) and (24), lay reasonably close to the respectively calculated curves. On the other hand, the point (*a*) corresponding to Eq (22), forms an ‘outlier’. This discrepancy can be attributed to the unusual total lack of male-male sharing as seen in Table 1. (see the levels of confidence in Fig 10).

**Fig 11:**
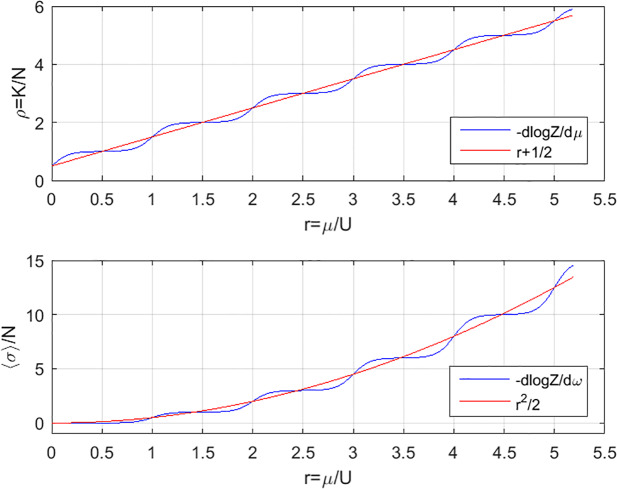
The density *ρ* = *K*/*N* and the average linkage per den *ξ* = 〈*σ*〉/*N* as a function of *r* = *μ*/*U* for large number of animals (1 < *N* ≪ *K*). In red—weak interaction, *ρ* = *r* + 1/2 and *ξ* = *r*^2^/2. In blue—strong repulsive interaction *U* ≃ 12.

Fig 10 demonstrates the tradeoff between gain by having a high female-male linkage and loss to a large number of outsiders. Thus, as one increases the density of animals, by reducing the number of pots, 〈*n*_0_〉 and 〈*σ*_*fm*_〉 start growing together and keep increasing monotonically, until reaching a turning-point (in our case, that point is specified as *N* = 2) where further increase of the density causes a decrease of 〈*σ*_*fm*_〉, accompanied by continuing increase of 〈*n*_0_〉. Fig 10 also presents the expected uncertainties in *n*_0_ and *σ*_*fm*_ which are essential for making comparison with experiments, especially for small systems [see Eq (13)]. The opposite case of a large system 1 ≤ *N* ≪ *K* is of particular interest. Referring to Eq (4) and setting *r* ≡ *μ*/*U*, we find that for weak interaction the density *ρ* ≡ *K*/*N* and the average linkage per den *ξ* ≡ 〈*σ*〉/*N* are smooth functions of *r*. However, as *U* increases (*U* ≃ 4*π*), *ρ* and *ξ* cross over to staircase-like curves (Fig 11) which resembles the Mott-Hubbard transition [39].

## Conclusion

In pioneering studies of “use of space”by the octopus *O. joubini*, the psychologist J. Mather characterized spatial distributions of octopus in the lab and in the wild [1, 7]. Minimal, if any, spatial organization was evident, a striking contrast to other cephalopods such as squid and certain species of cuttlefish that at least before maturity are often observed in schools [40, 41]. Mather studied densities of individuals, potential clustering indicative of spatial inhomogeneity, and measures of hierarchy/dominance and territoriality. As she observed, aggregation does not necessarily reflect interaction.

One of her principal conclusions can be recast in standard physics terminology as the finding that over characteristic scales exceeding a few octopus lengths, static spatial octopus distributions—although not homogeneous in the lab, where edge effects asserted themselves—are fully determined by single-particle correlations (one-body densities) and exhibit no order even at short ranges. That is, at least insofar as her measurements were concerned, observed densities of these live animals were indistinguishable from those of classical non-interacting particles in an external field, in this instance the local seafloor shell density that presumably reflected prey abundance.

Mather suggested [15, 30] that the term “asocial”is a suitable designation for this behavior wherein the animals ignore one another. Unfortunately, certain pivotal quantities such as mean free path and impact parameter that would pin down how often animals approached one another within a specified distance.

A pivotal, if implicit, contribution of Mather was her elucidation of a link between animals’ “use of space”- simplistically, their spatial organization, although temporal components may also be relevant—and “social behavior.”Spatial organization is measurable even when attribution of observed use of space specifically to social behavior—or even to animate agency—is uncertain, and may itself not be quantifiable. “Sociality”is an elusive concept; the difficulties that arise in trying to define it crystallize in robotics, for example, wherein inanimate objects can exhibit collective swarm-like behaviors [42]. Once living organisms are viewed as wetware machinery, the arbitrariness inherent to any particular definition of “sociality”is uncontroversial. Nevertheless, once one has in mind a specific purpose, definitions of sociality customized to achieve clearly articulated predictions of behavior on explicitly stated terms may become possible. Thus, as discussed at the 2018 Aspen Center for Physics workshop on ‘Physics of behavior’, any quantitative measure of ‘sociality’ is heavily dependent on context.

For these reasons, we revisited the ideas raised by Mather in a slightly different context and with modern quantitative tools. We were motivated by field observations of unanticipated behavior of a native Okinawan octopus that frequents coral reefs, *O. laqueus*. Individuals were observed anecdotally in our field expeditions to share dens, which cephalopod specialists found surprising for what they customarily regard as an asocial genus. We studied *O. laqueus* under laboratory configurations wherein we could vary the number of dens and octopus within a tank and directly measure how multiple occupancy depended on those values. We inferred parameters of a minimal model to maintain predictive value, and we painstakingly characterized uncertainty, so that our findings can in principle be invalidated. Our study is of potential importance for humane laboratory and industrial culture of cephalopods under conditions wherein they share a tank.

We aim to develop reproducible laboratory measures that reflect (and eventually predict) field observations that could be relevant for successful commercial culture of the animal. The field observations reported here of octopus *O. laqueus* engaging in den-sharing, a behavior which is thought to be atypical of most octopus species, could indicate that they are more readily cultured in the lab without cannibalization than are other species of octopus. Anecdotal evidence suggested that *O. laqueus* individuals tolerate one another: field observations of two animals apparently sharing the same den; the willingness of multiple individuals to cohabit indefinitely within a single tank without a lid, a condition wherein many octopus species would—in our experience—flee the tank to certain death in a dark corner of the lab. The challenge is to move beyond anecdote. As with all biological systems, experiments in the lab and their modeling often come at the cost of artificial or unnatural settings. Octopuses that are not well-fed, for example, may harm one another, but EU guidelines and animal welfare considerations preclude keeping octopus under conditions wherein they may be subject to harm.

Our anecdotal observation of den sharing in the field, first reported here, suggested to us that den sharing could be recast into a laboratory measure that might plausibly reflect certain aspects of sociality. In our hands, *O. laqueus* in laboratory tanks equipped with clay pots, exhibit distinctive behavior wherein they explore the dens in the morning hours before settling in for the day. Indeed, it is this observation—suggestive of ergodicity—that could account for the apparent validity of the equilibrium theory invoked here. Den sharing provides a readily measurable observable amenable to parameterization by number of dens and number of animals. Because our measure was crude, we were able to establish statistical uncertainty by assessing the *independence* of measurements with a suitably-defined *correlation time* without which statistical characterizations often performed in the literature on sociality are rendered meaningless.

We studied the social tolerance of *O. laqueus* by measuring the den occupancy of dens in the lab for varying densities of animals and several sex-mixtures. We found that *O. laqueus* tolerate other individuals by sharing tanks and dens, with typically no loss to cannibalism or escape. However, animals also exhibit significant levels of repulsion, and individuals often chose a solitary den when given the option. The patterns of den occupancy were studied with a maximum entropy model that treated animals as particles with on-site pair interaction. The three interaction parameters that determine the amount of social attraction/repulsion between animals according to sex, together with the chemical potential that confines animals to dens, were estimated from the experiment by a standard maximum likelihood calculation. The parameters computed in this way were then used to characterize the social behavior in large set of experimental conditions and to identify potential outliers. This procedure, as well as the general applicability of a maximum entropy model in this context, remain to be verified in future experiments with independently obtained or larger sample statistics.

## Supporting information

S1 Video Two *O. laqueus* in close proximity in the field, possibly sharing a den. (PDF)

S2 Video Elastomere injection of *O. laqueus*. (PDF)

S1 Table (PDF)

S2 Table (PDF)

S3 Table (PDF)

S4 Table (PDF)

S1 File (M)

S2 File (M)

S3 File (M)

S4 File (M)

S5 File (M)

S6 File (M)

S7 File (M)

S8 File (M)

S9 File (NB)

S10 File (NB)

S11 File (M)

S12 File (NB)

S13 File (NB)

S14 File (NB)

S15 File (NB)

S16 File (M)

S17 File (M)

S18 File (M)

S19 File (NB)

S20 File (DOCX)

S21 File (M)

S22 File (M)

S23 File (M)

S24 File (M)

S25 File (NB)

S26 File (NB)

S27 File (NB)

S28 File (NB)

S29 File (NB)

S30 File (NB)

S31 File (NB)

S32 File (NB)

S33 File (NB)

S34 File (NB)

S35 File (NB)

S36 File (NB)

S37 File (NB)

S38 File (M)
