# Supplementary material for: Social tolerance in Octopus laqueus—A maximum entropy model
Source: PLoS One. 2020 Jun 10;15(6):e0233834. doi: 10.1371/journal.pone.0233834 (PMC7286511; doi:10.1371/journal.pone.0233834)
Supplement: S4 Table — (PDF) [file pone.0233834.s006.pdf]

Male-Female 3:1 octopus:pots	Day	Tank 2 Outside Pots	Tank 2 Pot 1	Tank 2 Pot 2	Elastomer Pattern ID	Weight
Replicate 1	1	2-RG	2-O	2-GR	2-B	17.8
Tank 2			2-G	2-B	2-G	39.4
				2-R	2-GR	21.5
	2		2-RG	2-B	2-R	32.2
			2-GR	2-O	2-RG	22.9
				2-R	2-O	9
				2-G		
	3	2-RG	2-R	2-G		
			2-B	2-GR		
				2-O		
	4	2-RG	2-GR	2-G		
			2-B	2-R		
			2-O			
	5	2-RG	**2-G**	2-R		
			**2-GR**	2-B		
				2-O		
	6	2-RG	2-O	2-R		
		2-B	2-GR	2-G		
	7		2-G	2-GR		
			2-R	2-RG		
				2-B		
				2-O		
**Male-Female 3:1 octopus:pots**	**Day**	**Tank 4 Outside Pots**	**Tank 4 Pot 1**	**Tank 4 Pot 2**	**Elastomer Pattern ID**	**Weight**
Replicate 2	1	4-O little*	4-O big	4-B	4-B	26.8
Tank 4				4-R	4-G	19.1
				4-G	4-R	40.3
				4-RG	4-RG	36.6
	2	4-O little*	4-O	4-RG	4-O big	17.8
			4-B	4-G	4-O little *	2.5
				4-O big	4-OB	13.2
	3	4-O big	4-B	4-RG		
			4-OB	4-G		
			4-R			

		4-O big	4-R	4-B		
	4		4-G	4-OB		
				4-RG		
		4-O big	4-R	4-B		
	5		4-G	4-RG		
				4-OB		
		4-G	4-R	4-OB		
	6		4-B	4-RG		
			4-O big			**KEY**
		4-G	4-R	4-B		Female
	7		4-OB	4-RG		Male
				4-O big		Large 30+ grams
						Medium 15-30 grams
	* Octopus 4-O little replaced by octopus 4-OB after the second day due to tiny size of octopus 4-O little					Small 0-15 grams
