# Supplementary material for: Social tolerance in Octopus laqueus—A maximum entropy model
Source: PLoS One. 2020 Jun 10;15(6):e0233834. doi: 10.1371/journal.pone.0233834 (PMC7286511; doi:10.1371/journal.pone.0233834)
Supplement: S20 File — (DOCX) [file pone.0233834.s026.docx]

The (29) scripts that were used for generating all the computational results are given in Table S1 below. These are either MATLAB scripts (.m) Ver. 2015b, or Mathematica notebooks (.nb) Ver. 8.0.1.0. We have made the names of all scripts self-explanatory. For example, **tab9_case3p4.nb** is a Mathematica notebook used for calculating Table 9, row (3+4). The right column of Tab. S1 states which m-function(s) or Mathematica Package are called within a script. A short description of the m-functions and the Mathematica Package – OctinJar.nb is given in Table S2.

| Table S1 |  |
| --- | --- |
| Matlab scripts (m)/  Mathenmatica notebooks (nb) | Uses: |
| eqn_15.m | anovan.m |
| eqn_16a.m, eqn_16b.m | cfgs.m |
| eqn_18a.nb, eqn_18b.nb | OCTinJar.nb |
| eqn_20a.m | cfg.m,emp_stat.m |
| eqn_22.nb | OCTinJar.nb |
| eqn_23.nb | OCTinJar.nb |
| eqn_24.nb | OCTinJar.nb |
| eqn_25.nb | OCTinJar.nb |
|  |  |
| line_400.m | xcov.m |
| line_439.m | wtt.m |
| line_514.m | wtt.m |
|  |  |
| tab_5.m | t1test.m |
| tab_6.m | anovan.m |
| tab_8.m | cfg.m, degen.m |
| tab9_case1.nb, tab9_case2.nb | OCTinJar.nb |
| tab9_case1p2.nb | OCTinJar.nb |
| tab9_case3.nb, tab9_case4.nb | OCTinJar.nb |
| tab9_case3p4.nb | OCTinJar.nb |
| tab9_case5.nb, tab9_case6.nb | OCTinJar.nb |
| tab9_case5p6.nb | OCTinJar.nb |
| tab9_case7.nb, tab9_case8.nb | OCTinJar.nb |
| tab9_case7p8.nb | OCTinJar.nb |
| tab9_all_combined | OCTinJar.nb |
|  |  |

| Table S2 |  |
| --- | --- |
| Matlab m-functions  ver. 2015b | Description |
| C=cfg(N,K), cfgs(N,K) | Calculation of all configurations for K animals distributed in N jars. The size of matrix C is [ncfg,N] where ncfg=Binomial(N+K-1,K). |
| g=degen(K,C) | The multinomial degeneracy of a configuration {n0,n1,…,Nn}: K=(n0+n1+…+nN) is the number of animals. C[ncfg,N] is the configuration matrix. g= the multinomial degeneracy. |
| [J,S]=emp_stat[dat,C] | Empirical statistics: dat[M,N] are the occupation data of M days. Variable J with fields {tab,av,vr,sd,er}, stores the sample-statistics of links, namely, the tabulated values, average value, variance, std and estimated error). S are similar sample-statistics for the daily sharing levels. |
| [t1,p1]=t1test(x1,m1) | One-sample t-test: x1=data, m1=reference mean value. t1 is the t-statistic and p1 is the corresponding one-sided p-value. |
| [t,pval]=wtt(x1,x2,s1,s2,n1,n2) | Two-sample Welch-test: x1=sample #1 (size n1 and sample-std= s1).  x2=sample #2 (size n2 and sample-std= s2). t is the t-statistic and pval is he corresponding one-sided p-value. |
| [rej,fpr,tau0]=corr_time(xx,dt) | Correlation time of a temporal signal [Ref :J.A. Hanson and H. Yang, Jour. Chem. Phys. {\bf 128}, 214101 (2008)]  xx=data, dt=sampling-interval. tau0=estimated correlation time. |
| Mathematica package  Ver. 8.0.1.0 | OCTinJar.nb defines the following functions: |
| functions | Description |
| cfg[k,n] | All configurations for K animals distributed in N jars. |
| degen[cfg] | The multinomial degeneracies of the configuration matrix. |
| h1[cfg,t,mu,u,i] | The one-particle energy vector of the configuration-matrix cfg as a function of (mu,u) for 1<=i<=num[cfg].  t=1 if n0 is to be included in cfg. |
| h2[c1,t1,c2,t2,mu,uff,umm,ufm,i,j] | The two-particle energy matrix of (c1Xc2) as a function of (mu, uff,umm,ufm), for 1<=i<=num[c1],1<=j<=num[c2]. t1=1(t2=1) if n0 is included in c1(c2). |
