# Supplementary material for: Social tolerance in Octopus laqueus—A maximum entropy model
Source: PLoS One. 2020 Jun 10;15(6):e0233834. doi: 10.1371/journal.pone.0233834 (PMC7286511; doi:10.1371/journal.pone.0233834)
Supplement: S3 Table — (PDF) [file pone.0233834.s005.pdf]

Male-Female 1:1 octopus:pots	Day	Tank 1 Outside Pots	Tank 1 Pot 1	Tank 1 Pot 2	Tank 1 Pot 3	Tank 1 Pot 4	Tank 1 Pot 5	Tank 1 Pot 6	Elastomer Pattern ID	Weight
Replicate 1	1		1-GR	1-G	1-R	1-B	1-O	1-RG	1-B	21.3
Tank 1	2		1-O	1-RG	1-B	1-R	1-G		1-G	5
			1-GR						1-GR	21.1
	3		1-O	1-R	1-GR	1-G	1-B	1-RG	1-R	46.1
	4		1-R	1-RG	1-B		1-GR	1-O	1-RG	15.3
				1-G					1-O	31.7
	5		1-G	1-GR	1-R	1-RG	1-B	1-O		
	6		1-R	1-B	1-GR	1-G		1-RG		
								1-O		

Male-Female 1:1 octopus:pots	Day	Tank 3 Outside Pots	Tank 3 Pot 1	Tank 3 Pot 2	Tank 3 Pot 3	Tank 3 Pot 4	Tank 3 Pot 5	Tank 3 Pot 6	Elastomer Pattern ID	Weight
Replicate 2	1	3-O	3-RG		3-R		3-B	3-GR	3-B	17.8
Tank 3			3-G						3-G	23.6
	2			3-O	3-B	3-RG	3-G	3-GR	3-GR	25.3
							3-R		3-R	39.7
	3			3-G	3-B	3-RG	3-R	3-O	3-RG	32.9
							3-GR		3-O	4.5
	4		3-G	3-RG			3-B	3-R		
				3-GR				3-O		
	5		3-B	3-O	3-G			3-RG		
					3-R			3-GR		

KEY
Female
Male
Large 30+ grams
Medium 15-30 grams
Small 0-15 grams
